# Recent Advances in Metal Nanoclusters: From Novel Synthesis to Emerging Applications

**DOI:** 10.3390/molecules30193848

**Published:** 2025-09-23

**Authors:** Alexandru-Milentie Hada, Marc Lamy de la Chapelle, Monica Focsan, Simion Astilean

**Affiliations:** 1 Nanobiophotonics and Laser Microspectroscopy Centre, Interdisciplinary Research Institute on Bio-Nano-Sciences, Babeș-Bolyai University, Treboniu Laurian Street, 400271 Cluj-Napoca, Romania; alexandru.hada@ubbcluj.ro (A.-M.H.); marc.lamydelachapelle@univ-lemans.fr (M.L.d.l.C.); monica.iosin@ubbcluj.ro (M.F.); 2 IMMM—UMR 6283 CNRS, Le Mans Université, Olivier Messiaen Avenue, 72085 Le Mans, France; 3 Biomolecular Physics Department, Faculty of Physics, Babes-Bolyai University, Mihail Kogalniceanu Street, 400084 Cluj-Napoca, Romania

**Keywords:** metallic nanoclusters synthesis, photoluminescence, imaging, theranostic, sensing, catalysis

## Abstract

Metallic nanoclusters (NCs), composed of a few to a hundred atoms, occupy a unique space between molecules and nanoparticles, exhibiting discrete electronic states, strong photoluminescence, and size-dependent catalytic activity. Their ultrasmall cores (<3 nm) and ligand-controlled surfaces confer tunable optical, electronic, and catalytic properties, making them attractive for diverse applications. In recent years, significant progress has been made toward developing faster, more reproducible, and scalable synthesis routes beyond classical wet-chemical reduction. Emerging strategies such as microwave-, photochemical-, sonochemical-, and catalytically assisted syntheses, together with smart, automation-driven platforms, have improved efficiency, structural control, and environmental compatibility. These advances have accelerated the deployment of NCs in imaging, sensing, and catalysis. Near-infrared emitting NCs enable deep-tissue, high-contrast fluorescence imaging, while theranostic platforms combine diagnostic precision with photothermal or photodynamic therapy, gene delivery, and anti-inflammatory treatment. NC-based sensors allow ultrasensitive detection of ions, small molecules, and pathogens, and atomically precise NCs have enabled efficient CO_2_ reduction, water splitting, and nitrogen fixation. Therefore, in this review, we highlight studies reported in the past five years on the synthesis and applications of metallic NCs, linking emerging methodologies to their functional potential in nanotechnology.

## 1. Introduction

Metal nanoclusters (NCs), typically composed of a few to a hundred atoms, occupy a unique regime between discrete molecules and larger nanoparticles (NPs). Unlike conventional NPs, which display continuous electronic bands [1], NCs exhibit molecule-like properties, including quantized energy levels, HOMO–LUMO transitions, and distinct photoluminescence [2]. Their ultrasmall core size (typically < 3 nm) results in a high fraction of surface atoms and well-defined atomic configurations, granting them exceptional optical, catalytic, and electronic characteristics that are highly sensitive to size, core composition and capping ligands, and surface environment. This atomic precision allows NCs to outperform traditional NPs in many applications where tunability, reproducibility, or biocompatibility are essential.

A defining feature of metal NCs is their strong and tunable photoluminescence, which arises from their discrete electronic states and ligand–metal interactions [3]. Several emission mechanisms have been proposed to account for the observed fluorescence in NCs, depending on their composition, size, and surface chemistry. In many cases, photoluminescence is attributed to ligand-to-metal charge transfer (LMCT) or ligand-to-metal–metal charge transfer (LMMCT), particularly in thiolate- or protein-protected NCs [4,5]. These mechanisms involve excitation-induced electron transfer from the surface ligands to the metal core or between metal atoms modulated by the ligand shell, followed by radiative recombination [6]. In some systems, phosphorescence-like behavior with long lifetimes and large Stokes shifts suggests involvement of triplet states or surface-state-mediated emission [7,8,9]. More recently, intersystem crossing and aggregation-induced emission (AIE) effects have also been identified [10,11,12,13,14]. These diverse mechanisms reflect the complex interplay between cluster core, surface ligands, and environmental factors, and are still the subject of active research aimed at achieving better control and predictability of NC luminescence for targeted applications.

Traditionally, the synthesis of metal NCs has relied on wet chemical reduction methods [15], where metal salts are reduced in the presence of protecting ligands under mild thermal conditions. While this classical approach is straightforward and adaptable to different ligands and metal precursors, it often suffers from long reaction times, limited control over cluster size and composition, poor reproducibility, and challenges in scaling up. As the demand for NCs in real-world applications grows, it becomes increasingly important to develop more efficient, reproducible, and scalable synthesis routes that preserve atomic precision while improving throughput and structural uniformity.

To address these challenges, this review focuses on emerging synthetic strategies, published in the last 5 years, that accelerate NC production and improve structural control. We discuss recent advances in microwave-assisted, photochemical, sonochemical, catalytically assisted, and smart synthesis approaches, each offering unique advantages in terms of speed, scalability, and structural precision. Beyond synthesis, we also highlight key applications where these newly developed NCs have demonstrated exceptional functionality. Specifically, we focus on their roles in fluorescence imaging, theranostics, chemical and biological sensing, and photocatalytic transformations. The main emphasis is placed on gold (Au), silver (Ag), and copper (Cu) NCs, as these remain the most widely explored systems. However, where relevant, examples of other metallic NCs such as platinum, palladium, and alloyed systems are also included to provide a broader perspective. Regarding stabilizers, this review primarily considers organic ligands such as proteins, peptides, and amino acids, while excluding inorganic stabilizing matrices such as glasses or zeolites.

The aim of this review is to provide an integrated perspective that connects recent synthetic innovations with their functional outcomes, offering researchers a clear view of the current state and future potential of metallic NCs.

## 2. Novel Synthesis

The synthesis of metal NCs traditionally relies on solution-phase chemical reduction under mild conditions, often involving prolonged stirring and heating in the presence of stabilizing ligands [16,17]. This classical approach, typically carried out in aqueous or organic solvents without specific templating strategies, offers a simple and accessible route to NC formation. However, it suffers from several drawbacks, including long reaction times (ranging from hours to several days), limited size control, and poor reproducibility due to sensitivity to reaction conditions. Emerging methodologies such as microwave-assisted, photochemical, sonochemical, and catalysis-assisted syntheses have significantly reduced reaction times and enhanced uniformity. More recently, the concept of “smart synthesis” has gained traction, leveraging machine learning algorithms, automation, and robotic platforms to optimize reaction parameters and accelerate the discovery and design of NCs with tailored properties.

### 2.1. Microwave-Assisted Synthesis

Microwave-assisted synthesis has emerged as a powerful alternative to conventional heating methods for the preparation of metal NCs. In this approach, rapid volumetric heating generated by microwave radiation accelerates the reduction in metal precursors and promotes uniform nucleation and growth. This technique offers significant advantages over classical methods, including substantially reduced reaction time, improved reaction reproducibility, and enhanced control over cluster size and emission properties. Moreover, it is inherently compatible with green chemistry principles, often enabling aqueous-phase, ligand-assisted syntheses without the need for harsh reductants or elevated temperatures [18].

Recent advances highlight the broad applicability of microwave-assisted synthesis for various metal NCs, including those based on Au, Ag, and Cu. For instance, our group has explicitly demonstrated the effect of microwave-assisted synthesis on the luminescence efficiency of histidine-stabilized AuNCs [19]. We reported that NCs synthesized via microwave heating (850 W, 30 min) exhibited fourfold higher photoluminescence than their counterparts obtained through classical, room-temperature protocols. 

**Figure 1:**
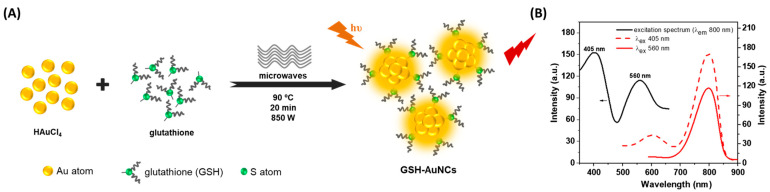
(**A**) Schematic illustration of the one-step synthesis process of glutathione-stabilized photoluminescent AuNCs. (**B**) Excitation (λ_em_ at 800 nm) and photoluminescence spectra (λ_ex_ at 405 and 560 nm) of GSH-AuNCs. Reprinted with permission from Ref [21]. Copyright 2022 Springer.

The clusters showed a single blue emission band centered at 471 nm under 380 nm excitation, along with excellent photostability and stability over time. This study provided clear experimental evidence of the enhanced optical quality and consistency afforded by microwave synthesis. In another work, we also demonstrated that microwave-assisted synthesis enables fine control over the structure and emission of solid-state histidine-stabilized AuNCs [20]. We employed the same microwave-assisted protocol (850 W, 90 °C, 30 min) to synthesize colloidal histidine-stabilized AuNCs, which were subsequently lyophilized to obtain solid-state materials. These NCs exhibited dual emission at 475 nm and 520 nm depending on excitation wavelength (340–520 nm), along with average fluorescence lifetimes of 2–3 ns. Notably, the emission remained stable under continuous UV irradiation and was preserved up to 150 °C, reflecting both photostability and thermostability. The excitation-dependent dual emission and robust performance were attributed to enhanced core–ligand interactions and increased structural uniformity induced by microwave heating. In a related effort, our group also employed a rapid microwave-assisted synthesis of AuNCs using glutathione (GSH) as a stabilizing ligand [21]. The reaction, conducted in sealed microwave vessels at 90 °C and 850 W for 20 min, yielded dual-emissive AuNCs with distinct photoluminescence peaks in the red (610 nm) and near-infrared (800–810 nm) regions (Figure 1).

The clusters could be excited over a broad spectral window (405–640 nm), with especially strong emission responses at the spectral edges. A high quantum yield of 9.9% was achieved, along with long fluorescence lifetimes, 407 ns in the red and up to 1821 ns in the NIR range, indicative of a well-passivated, rigid NC structure. These photophysical characteristics were directly enabled by the rapid and homogeneous reduction in Au precursors under microwave irradiation, further reinforcing the advantages of this method for producing structurally robust and optically tunable NCs.

Beyond gold-based systems, microwave-assisted protocols have also proven effective for other metals. A representative example is the work of Shang et al., who developed a rapid and environmentally friendly synthesis of AgNCs using L-histidine as both the reducing and stabilizing agent [22]. Specifically, a mixture of AgNO_3_ and histidine was subjected to microwave irradiation at 700 W for only eight minutes, resulting in the formation of Ag^0^-core NCs with surface-bound Ag^+^ coordinated to histidine. The clusters exhibited strong blue fluorescence with an emission peak at 440 nm upon 356 nm excitation, a quantum yield of 5.2%, and a multiexponential fluorescence decay with an average lifetime of 5.06 ns. The rapid formation, absence of harsh chemicals, and control over optical output were direct benefits of the microwave-assisted approach. This strategy has also been successfully extended to Cu-based systems, which present additional synthetic challenges due to their susceptibility to oxidation. In this context, Saleh et al. reported the microwave-assisted synthesis of pepsin-stabilized CuNCs by irradiating a basic aqueous solution of Cu(NO_3_)_2_ and pepsin at 850 W for 30 min [23]. The obtained pepsin-stabilized CuNCs exhibited a uniform size of approximately 2 nm. These clusters emitted blue fluorescence with an emission maximum at 409 nm upon 360 nm excitation and exhibited an impressive quantum yield of 17%, one of the highest reported for aqueous CuNCs. The efficiency of the microwave-assisted approach in this case was attributed to rapid nucleation and controlled reduction, which helped mitigate oxidative degradation and produced stable, luminescent NCs.

In conclusion, microwave-assisted synthesis has demonstrated significant potential as a rapid, reproducible, and green route for the preparation of luminescent metal NCs. The microwave-assisted strategy exhibits broad applicability across Au, Ag, and Cu systems, with consistent improvements in reaction time, optical performance, and structural stability. By enabling efficient precursor reduction and uniform NC formation, microwave irradiation allows fine control over emission characteristics. These advantages underscore the value of microwave-assisted protocols as a modern alternative to classical synthesis. However, microwave-assisted synthesis is not without limitations. One important challenge is its poor cross-laboratory reproducibility, as reaction outcomes are highly sensitive to experimental conditions. In particular, the required power and irradiation time can vary significantly depending on the model of the microwave oven and the reaction vessel volume. These factors must be carefully considered and standardized to ensure reliable and reproducible results. Nevertheless, despite these challenges, microwave-assisted synthesis generally remains a faster and more efficient alternative to conventional heating methods.

### 2.2. Photochemical-Assisted Synthesis

Photochemical-assisted synthesis has emerged as a powerful method for producing metal NCs under mild, controllable, and environmentally friendly conditions. In contrast to conventional chemical reductions, photochemical strategies utilize light to drive redox transformations, enabling precise temporal control over nucleation and growth. This approach offers several advantages: it can bypass uncontrolled reduction kinetics, minimize the use of hazardous reagents, and, in some cases, generate unique structural features or oxidation states that are difficult to achieve through thermal or chemical methods. The light-triggered mechanisms can operate via direct photoreduction, photoinduced electron transfer (PET), or radical-mediated redox cascades, depending on the metal system and ligand environment.

**Figure 2:**
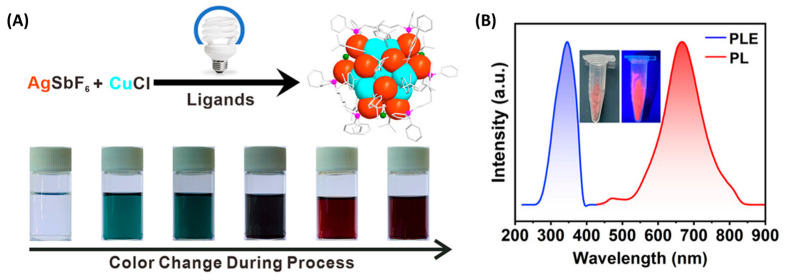
(**A**) Illustration of the photochemical synthesis of Ag_12_Cu_7_. Atom color scheme: orange—Ag, turquoise—Cu, pink—P, green—Cl, gray—C. Hydrogen atoms are omitted for clarity. (**B**) Excitation and photoluminescence spectra of the Ag_12_Cu_7_. Reprinted with permission from Ref. [25]. Copyright 2024 John Wiley and Sons.

A representative example is the work of Wang et al., who synthesized atomically precise Ag_25_ NCs using a PET mechanism under white LED irradiation [24]. Triethylamine acted as a sacrificial donor, transferring electrons to Ag^+^ to initiate cluster growth, with O_2_ as the terminal oxidant. Notably, this method required no chemical reductants and allowed structural control comparable to that achieved using NaBH_4_, but under greener, milder conditions. Building on this, the same group developed a stepwise synthesis of a bimetallic Ag_12_Cu_7_ cluster [25]. Ag_19_ was first photochemically generated, followed by CuCl addition under continued irradiation (Figure 2).

This sequential reduction overcame redox incompatibility between Ag^+^ and Cu^+^ and enabled controlled alloying. The final cluster exhibited deep red phosphorescence (λ_em_ = 665 nm) with a 30 µs lifetime at room temperature, attributed to strong cuprophilic interactions and ligand-to-metal charge transfer.

Photochemical methods have also enabled access to well-defined group 10 metal NCs. Fan et al. synthesized for the first time nickel (Ni) and palladium (Pd) clusters via a redox cascade triggered by blue light [26]. After NaBH_4_ pre-reduction, irradiation generated thiyl radicals from disulfides, which stabilized metal aggregates into discrete ring-like structures such as Ni_11_(SPh)_22_. This approach succeeded where conventional reductions failed, offering better crystallinity, stability, and unique geometries. UV-Vis analysis revealed a characteristic absorption peak at 467 nm for Ni_11_. In a sustainable chemistry context, Ferlazzo et al. reported a green photochemical synthesis of CuNCs in water using UV-activated acetone and monoethanolamine (MEA) as stabilizer [27]. The resulting clusters (~3.5 nm) showed blue-green emission centered at 489.9 nm, a quantum yield of 12%, and excellent stability. They also exhibited photothermal behavior under 405 nm laser irradiation, with 38% conversion efficiency. The method avoided toxic solvents and reducing agents, emphasizing photochemistry’s role in green nanomaterials development. Therefore, photochemical-assisted synthesis provides a green and controllable route to metal NCs, enabling precise structural and optical tuning without harsh reductants. From noble to transition metals, this approach has proven effective across diverse systems, highlighting its growing importance in sustainable nanomaterial development.

### 2.3. Sonochemical-Assisted Synthesis

Sonochemical-assisted synthesis has emerged as a powerful technique for the fabrication of metal NCs, leveraging the physical and chemical effects of ultrasound to drive and control nucleation processes. When ultrasonic waves pass through liquid media, they generate acoustic cavitation, rapid formation and implosion of microbubbles, which produce localized hotspots with extreme temperature and pressure gradients. These transient conditions enhance mixing, accelerate reduction reactions, and promote uniform nucleation, often leading to the formation of smaller, more monodisperse clusters compared to conventional synthesis [28]. Furthermore, sonochemical processes can activate surfaces, assist ligand integration, and improve metal-support interactions.

**Figure 3:**
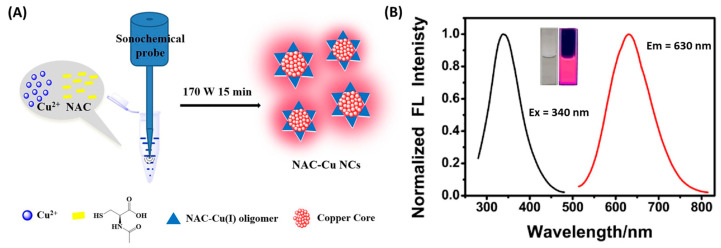
(**A**) Illustration of the sonochemical method used to synthesize NAC-stabilized CuNCs. (**B**) The excitation and photoluminescence spectra of the NAC-CuNCs. Reprinted with permission from Ref. [29]. Copyright 2022 Elsevier.

A clear demonstration of these advantages is provided by Kang et al., who synthesized red-emissive CuNCs stabilized by N-acetyl-L-cysteine (NAC) using a 15 min ultrasound-assisted method in water, without any external reducing agents (Figure 3) [29].

The process involved Cu^2+^ reduction to Cu^+^ and subsequent ultrasound-driven reduction to Cu^0^, resulting in ultrasmall clusters (~1.2 nm core size) with a red emission peak at 630 nm and a large Stokes shift of 290 nm. Compared to conventional thermal synthesis (12 h at 70 °C), the sonochemical approach produced brighter, more photostable NCs that remained stable for months. This synthesis strategy offered not only speed and simplicity but also enabled efficient integration of the NAC ligand, which functioned dually as reductant and stabilizer. The resulting NCs showed high luminescence lifetime (451 ns average) and excellent thermal and colloidal stability, confirming the effectiveness of ultrasound in driving clean, green synthesis with enhanced optical properties. Beyond Cu-based systems, sonochemical methods have also been employed to precisely control the nucleation and growth of transition metal NCs. Liu et al. developed an ultrasound-assisted route to synthesize nickel NCs supported on Ti_3_C_2_Tₓ MXene, where ultrasonic irradiation played a dual role in promoting MXene exfoliation and enabling uniform NiNC formation [30]. Compared to non-sonicated controls, the ultrasound-treated catalysts exhibited higher dispersion, smaller particle sizes, and a sixfold increase in turnover frequency (302 h^−1^). The synergy between ultrasound-induced surface activation and enhanced nucleation yielded electron-rich, catalytically active NiNCs with excellent stability. In summary, sonochemical-assisted synthesis offers a rapid, scalable, and environmentally friendly approach for producing well-defined metal NCs. By exploiting the localized energy of acoustic cavitation, this method enables precise control over nucleation, particle size, and ligand incorporation. Its versatility across different metal systems highlights its growing relevance in the development of high-performance nanomaterials under mild and sustainable conditions.

### 2.4. Catalytic-Assisted Synthesis

Catalytically assisted synthesis has recently emerged as a promising approach for generating atomically precise metal NCs under mild and selective conditions. Unlike traditional chemical reduction methods, catalytic systems can activate molecular hydrogen or other reductants to drive controlled metal ion reduction. These methods leverage well-established catalytic principles, such as hydrogen activation, proton-coupled electron transfer (PCET), and surface-mediated reactions, to facilitate NC formation in a tunable and potentially scalable manner. By integrating heterogeneous catalysts into NC synthesis, this strategy also opens the door for cleaner, recyclable, and industrially relevant processes.

A recent study by Wang et al. demonstrated the first heterogeneous catalytic synthesis of an atomically precise Au_13_ NC using commercial Pd/C and molecular hydrogen [31]. The Au precursor, (CH_3_)_2_SAuCl, was reduced in ethanol under 0.1 MPa H_2_ at room temperature, in the presence of thiol (TBBT), phosphine (Dppe) ligands, and a base (Et_3_N). The resulting cluster, PPh_4_[Au_13_(TBBT)_4_(Dppe)_4_]Br_2_, featured a centered icosahedral Au_13_ core stabilized by a mixed ligand shell and confirmed via single-crystal XRD. Control reactions showed that neither heat nor H_2_ alone could drive the transformation; Pd/C was essential to activate H_2_ and initiate reduction. Mechanistically, Pd–H species facilitated PCET to reduce Au(I), with Et_3_N lowering the energy barrier, as supported by DFT studies. The synthesis produced no aggregation, and the Pd/C catalyst was recoverable, highlighting the process’s selectivity and practicality. This work establishes catalytic hydrogenation as a viable, sustainable pathway for NC synthesis. Merging principles from heterogeneous catalysis and nanochemistry opens new possibilities for precise cluster construction under ambient and scalable conditions.

### 2.5. Smart Synthesis

Smart synthesis represents a next-generation strategy for preparing metal NCs, characterized by the integration of automation, intelligent feedback systems, and data-driven control over reaction variables. Unlike conventional methods, smart synthesis incorporates real-time monitoring, machine learning (ML), robotic systems, and high-throughput experimentation to optimize cluster formation with unprecedented precision [32]. This approach addresses long-standing challenges in NC synthesis, such as low reproducibility, sensitivity to minor changes in reaction conditions, and difficulty in navigating multidimensional parameter spaces, by enabling dynamic, adaptive control and autonomous decision-making.

**Figure 4:**
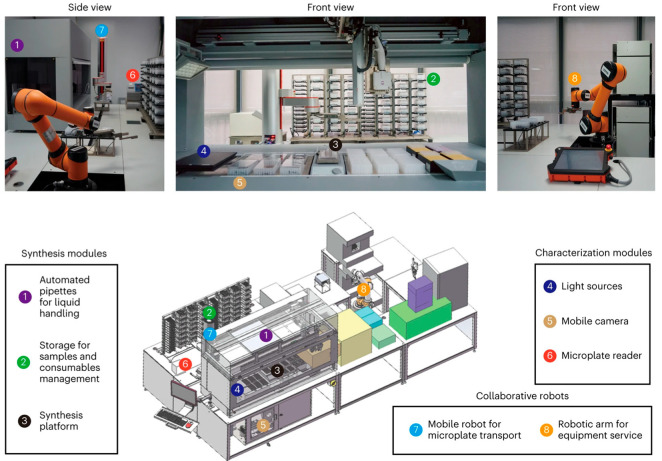
Robotic platform for the automated synthesis and characterization of NCs. Reprinted with permission from Ref. [33]. Copyright 2023 Elsevier.

Recently, a self-driving robotic workstation [33,34,35], guided by a closed-loop optimization algorithm, has been employed to synthesize nanocrystals. The robot autonomously adjusted parameters such as stirring speed and precursor concentration based on feedback from real-time UV–vis absorption and fluorescence spectra, rapidly converging on optimal conditions (Figure 4).

Moreover, ML-assisted reaction optimization has been used to tune ligand–metal ratios, pH, temperature, and reducing agent strength in real time, leading to the efficient identification of conditions that yield atomically precise clusters [36,37]. Importantly, smart synthesis not only accelerates discovery but also enhances reproducibility by minimizing human intervention. It facilitates the exploration of vast compositional and kinetic spaces that are often inaccessible through manual methods. While still in its early stages, the convergence of chemistry with robotics, data science, and artificial intelligence promises to redefine how NCs are discovered and manufactured. As these technologies mature, smart synthesis is expected to shift NC preparation from artisanal practice to programmable science, enabling scalable, reproducible, and application-specific design of NCs.

Taken together, these emerging methodologies demonstrate how alternative energy inputs and catalytic principles can overcome the limitations of conventional chemical reduction, offering faster, greener, and more reproducible access to metallic NCs. While the choice of synthesis route is essential for improving efficiency, scalability, and uniformity, it is the ligand chemistry and cluster size that remain the dominant factors in defining photoluminescence properties and functional performance. To provide a consolidated view of these advances, a summary of representative NCs synthesized via microwave-, photochemical-, sonochemical-, catalytically assisted, and smart approaches is presented in Table 1.

Nevertheless, purification of metallic NCs after synthesis is critical for obtaining well-defined systems with uniform nuclearity, ligand stoichiometry, and reproducible optical or catalytic properties. Common purification techniques include ultrafiltration or centrifugal membrane filtration devices (such as Vivaspin^®^) and dialysis/diafiltration, which remove excess ligands, salts, and low-molecular-weight byproducts. Polyacrylamide gel electrophoresis (PAGE) and its micro-preparative variant (MP-PAGE) are also widely employed, beyond serving as preparative tools to isolate specific NC species, they additionally provide an analytical readout of sample purity by showing whether a single or multiple bands are present. Chromatographic methods are frequently applied as well: size-exclusion chromatography (SEC) effectively removes aggregates or broad size distributions, while anion-exchange chromatography (AEC) and other high-performance liquid chromatography (HPLC) approaches allow fine discrimination between NCs of very similar size or ligand environments.

Characterization of purified NCs should always accompany purification, as understanding their structure–property relationships is essential for guiding applications. Ultraviolet–visible (UV-Vis) absorption spectroscopy and photoluminescence spectroscopy are the most commonly employed techniques, providing direct information about the electronic structure and optical properties of NCs. Band sharpness, emission wavelength, quantum yield, and excited-state lifetimes are key parameters that determine performance in fluorescence imaging, sensing, and optoelectronic applications. To gain deeper mechanistic insight into the excited-state dynamics, time-resolved PL spectroscopy can distinguish between prompt and delayed emission pathways, while transient absorption spectroscopy can capture ultrafast charge carrier relaxation and energy transfer processes.

Complementing these, transmission electron microscopy (TEM) and high-resolution TEM (HRTEM) provide insight into the core size, morphology, and dispersion state of NCs, which directly influence emission efficiency, catalytic activity, and biological distribution. X-ray photoelectron spectroscopy (XPS) probes surface chemistry, offering valuable information about oxidation state, ligand binding, and elemental composition of the outer shell. These properties are particularly relevant for stability, sensing interfaces, and catalytic reactivity. To confirm atomic composition and ligand stoichiometry with high precision, electrospray ionization mass spectrometry (ESI-MS) or matrix-assisted laser desorption/ionization mass spectrometry (MALDI-MS) is indispensable, enabling the identification of nuclearity and molecular formula of the clusters. PAGE, MP-PAGE, and HPLC serve both as purification and as diagnostic tools, allowing researchers to assess sample homogeneity and to separate distinct cluster populations when present. Finally, inductively coupled plasma mass spectrometry (ICP-MS) or atomic absorption spectroscopy (AAS) provides quantitative information on residual ionic content or metal impurities, which is crucial for evaluating biosafety and ensuring catalytic activity arises from NCs rather than from free ions.

In addition to these widely adopted methods, several underexplored spectroscopies can significantly advance mechanistic understanding. Electron paramagnetic resonance (EPR) spectroscopy can provide direct evidence of radical intermediates or paramagnetic states involved in photophysical or catalytic pathways. Nuclear magnetic resonance (NMR) spectroscopy remains underutilized but is highly informative for probing ligand–metal interactions and dynamic ligand exchange. Moreover, Raman spectroscopy can selectively enhance vibrational modes coupled to electronic transitions, offering insight into metal–ligand charge transfer states. Integrating these advanced techniques with conventional optical and structural characterization will be critical to move beyond phenomenological description toward a molecular-level mechanistic understanding of NC emission and reactivity.

## 3. Applications

### 3.1. Fluorescence Imaging

Metal NCs have emerged as highly promising fluorescent probes for bioimaging due to their ultra-small size, high photostability, tunable emission, and excellent biocompatibility [38]. Their discrete electronic states and strong ligand–metal interactions enable bright and stable luminescence, making them attractive candidates for applications where traditional organic dyes or quantum dots fall short. Although fluorescent NCs have been developed across the visible to near-infrared (NIR) spectrum, this subchapter focuses on those emitting in the NIR region. Compared to visible-emitting agents, NIR-emitting NCs offer deeper tissue penetration, reduced background autofluorescence, and improved signal-to-noise ratios, all of which are critical for high-resolution imaging in biological environments [39]. Moreover, their compatibility with clinically relevant optical windows makes NIR-emitting NCs particularly attractive for translation into medical imaging applications. To highlight the versatility and translational potential of NCs, we examine their application across different biological settings, ranging from cellular models to tissue-level imaging and whole-organism studies. Accordingly, the following sections are organized into in vitro, ex vivo, and in vivo investigations, reflecting the increasing complexity and physiological relevance of each approach.

#### 3.1.1. In Vitro Fluorescence Imaging

In Vitro imaging studies serve as the foundational step in evaluating the bioimaging potential of NCs, enabling controlled investigation of their behavior in cellular environments. These experiments are critical for assessing cellular uptake, intracellular localization, cytotoxicity, and fluorescence performance under biologically relevant conditions. Moreover, when NCs are functionalized with targeting ligands, such as peptides, antibodies, aptamers, or small molecules, they can selectively bind to specific cell types, allowing precise visualization of pathological versus healthy cells. This makes in vitro imaging especially powerful for validating targeted NC designs, optimizing ligand–receptor interactions, and establishing molecular specificity before translation to tissue or whole-organism models.

**Figure 5:**
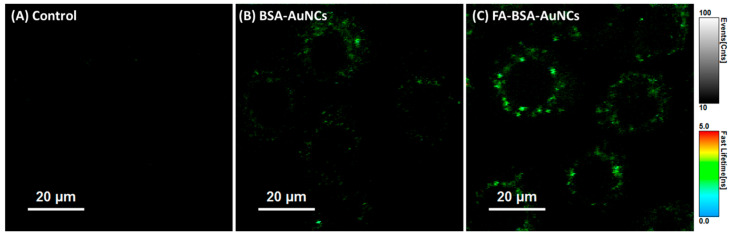
FLIM images of NIH:OVCAR-3 cells: (**A**) untreated control, (**B**) cells treated with BSA-AuNCs, and (**C**) cells treated with FA-conjugated BSA-AuNCs. Reprinted with permission from Ref. [40]. Copyright 2020 Elsevier.

One illustrative example was reported by our group and involves the use of folic acid (FA)-functionalized bovine serum albumin (BSA) stabilized AuNCs for targeted imaging of ovarian cancer cells [21]. These NCs, emitting at 670 nm under 530 nm excitation, were designed to exploit the overexpression of folate receptor alpha (FRα) on NIH:OVCAR-3 cells. FA was covalently linked to the surface of the BSA-AuNCs using EDC/NHS chemistry (1-ethyl-3-(3-dimethylaminopropyl)carbodiimide/ N-hydroxysuccinimide), resulting in a measurable increase in hydrodynamic size and zeta potential shift, confirming successful conjugation. Epi-fluorescence microscopy revealed intense cytoplasmic accumulation in FRα-positive cells, while confocal fluorescence lifetime imaging microscopy (FLIM) demonstrated enhanced contrast and lifetime-based discrimination from background autofluorescence (Figure 5).

Notably, the functionalized NCs showed stronger internalization and perinuclear accumulation compared to their non-targeted counterparts, without causing cytotoxic effects at high concentrations. Another approach to achieve cell specificity was demonstrated by Feng et al., who developed thiolated tumor-specific mucin 1 (MUC1) aptamer-stabilized AuNCs, where the DNA aptamer served simultaneously as a stabilizer and a recognition ligand [41]. The resulting NCs, measuring ~1.5 nm in diameter, exhibited strong red fluorescence at 655 nm under 420 nm excitation and a remarkably long fluorescence lifetime of 5.6 µs. Targeting specificity was validated by incubating the NCs with MUC1-positive 4T1 breast cancer cells and MUC1-negative 293T kidney cells. Only the 4T1 cells displayed bright cytoplasmic fluorescence, with signal intensity increasing over time. Lifetime imaging also confirmed that photoluminescence originated from specific interactions within the targeted cancer cells, rather than from passive or nonspecific uptake. This result highlights the strong selectivity of the aptamer-guided NCs for imaging MUC1-expressing tumor cells. Extending the range of targeting mechanisms, Tan et al. reported the synthesis of spider venom–derived peptide-stabilized AuNCs (LAuNCs) using lycosin-I, an amphiphilic and cationic peptide known for its tumor-penetrating capabilities [42]. These LAuNCs displayed far red emission at 682 nm upon 347 nm excitation, with a quantum yield of 9.1% and an average luminescence lifetime of 2.1 µs. Upon incubation with cancer cells (4T1 and A549), confocal microscopy revealed a two-stage process: initial cytoplasmic localization followed by gradual nuclear translocation over 8 h. This behavior was driven by intracellular GSH, which triggered the disassembly of peptide-induced aggregates into ultrasmall clusters, facilitating nuclear entry. Uptake was highly selective for cancer cells, with >94% internalization in 4T1 cells and negligible uptake in non-cancerous Hek293t cells, as confirmed by flow cytometry and Inductively Coupled Plasma Mass Spectrometry (ICP-MS) quantification.

These studies collectively highlight the versatility of NCs in achieving selective cellular imaging through rational surface functionalization. By employing diverse targeting strategies, from small molecules and aptamers to bioactive peptides, precise discrimination between cancerous and non-cancerous cells was achieved. The ability to tailor emission wavelengths, enhance photostability, and exploit long fluorescence lifetimes further positions NCs as powerful tools for high-contrast, background-free imaging at the cellular level. Such in vitro investigations lay a critical foundation for advancing targeted NC systems toward more complex biological applications.

#### 3.1.2. Ex Vivo Fluorescence Imaging

Ex Vivo imaging serves as an essential intermediate step between in vitro validation and in vivo translation, offering a more realistic assessment of NC performance in complex tissue environments. This approach allows for detailed evaluation of tissue penetration, signal retention, and contrast enhancement under near-physiological conditions. Importantly, ex vivo studies can also be conducted using artificial tissue-mimicking phantoms, providing a safe and reproducible platform to optimize imaging parameters and assess depth-resolved fluorescence performance before proceeding to animal models.

**Figure 6:**
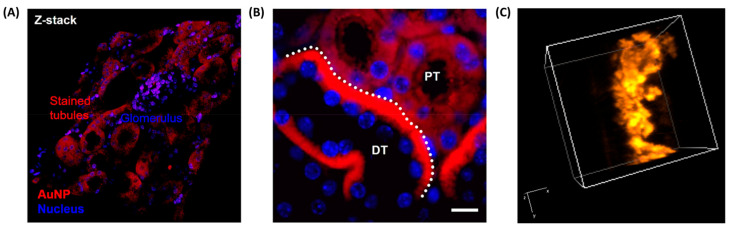
(**A**) Confocal fluorescence z-stack image of the kidney cortex showing localized GS-AuNPs (field width: 320 µm). (**B**) Microscopy image of kidney tissue sections (4 µm thick) displaying in situ formed ultrasmall GS-AuNPs. Scale bar: 10 µm. Abbreviations: PT—proximal tubule; DT—distal tubule. Reproduced from ref [43] with permission from American Chemical Society, Copyright 2019. (**C**) 3D Re-scan confocal microscopy (RCM) images of agarose phantom embedded with GSH-AuNCs (λₑₓ = 640 nm). Image Width 108.02 µm, Image Height 108.02 µm and Image Depth 100.00 µm. Reprinted with permission from Ref. [21]. Copyright 2022 Springer.

A notable example of selective localization in real tissue slices was demonstrated by Peng et al., who introduced an in situ ligand-directed synthesis approach for growing AuNCs directly within tissue sections [43]. Using biological ligands such as GSH or β-glucose-SH, Au precursors were selectively reduced within specific compartments of mouse kidney, brain, and intestinal tissue slices. This method led to ultrasmall, NIR-luminescent clusters during the early formation phase, with emission peaks at 730 and 800 nm under 350 nm excitation. Targeted accumulation was guided by natural ligand-tissue affinity; GSH promoted mitochondrial labeling in renal and neuronal regions, while β-glucose enabled selective labeling of the intestinal brush border (Figure 6A,B). Confocal microscopy, TEM, and whole-tissue NIR imaging confirmed the high spatial precision and multiscale imaging capability, establishing this method as a powerful histological mapping tool.

Complementing real tissue imaging, our group has demonstrated the utility of synthetic tissue-mimicking phantoms for evaluating NCs prior to in vivo application. These phantoms, composed of agarose, intralipid, and hemoglobin, replicate the scattering and absorption characteristics of biological tissues and serve as platforms for optimizing contrast agents. For instance, our group investigated the photophysical performance of BSA-stabilized AuNCs (BSA-AuNCs) in both solid-state form and in phantom matrices designed to simulate tumor-like optical properties [44]. The NCs were incorporated into agarose-based phantoms doped with intralipid and hemoglobin to mimic biological scattering and absorption. Under two-photon excitation fluorescence lifetime imaging microscopy (TPE–FLIM) at 810 nm, the NC-containing phantoms exhibited strong, homogeneous fluorescence and lifetime profiles that remained well-separated from background autofluorescence. These results validated the stability and imaging capacity of BSA-AuNCs in three-dimensional, tissue-like environments. Building on this, a second study from our group optimized the embedding concentration and imaging depth using colloidal BSA-AuNCs dispersed in similar phantom matrices [45]. By varying the volume fraction of NCs and performing TPE–FLIM at excitation wavelengths between 780 and 820 nm, we identified the optimal loading, yielding bright NIR emission without aggregation. Quadratic power dependence of the emission confirmed genuine two-photon excitation, while the lifetime mapping showed high contrast against the phantom background. Notably, imaging at 820 nm provided slightly higher signal intensity, reinforcing its suitability for deep-tissue and surgical guidance applications using BSA-AuNCs as contrast agents. Expanding on this, our group explored the embedding of dual-emissive GSH-AuNCs within phantoms to exploit both red (610 nm) and NIR (800 nm) emission channels for flexible imaging strategies [21]. These NCs, synthesized via a microwave-assisted method, displayed exceptionally long luminescence lifetimes up to 1.8 µs. This is a significant advantage for FLIM because it provides a clear contrast against the much shorter lifetimes (typically nanoseconds) of native biological fluorophores and tissue autofluorescence. This distinct temporal separation allows for selective imaging of the NCs, making them highly effective as FLIM contrast agents for detailed biological studies. Re-scan confocal microscopy (RCM) and FLIM confirmed their even distribution throughout the phantom and excellent signal separation from background autofluorescence (Figure 6C), which typically has lifetimes under 4 ns. This demonstrated the value of dual-mode (spectral and lifetime) contrast for tissue-relevant imaging platforms.

These findings highlight the suitability of metal NCs as powerful contrast agents for ex vivo fluorescence imaging. Their stable photoluminescence, tunable emission in the NIR region, and long fluorescence lifetimes enable high-contrast, background-free visualization in both real tissues and synthetic phantoms. Whether grown in situ within biological slices or embedded into scattering tissue models, NCs retain their optical integrity and targeting precision, making them ideal tools for optimizing imaging parameters and validating probe performance ahead of in vivo deployment.

#### 3.1.3. In Vivo Fluorescence Imaging

In vivo imaging represents the ultimate test for evaluating the real-world applicability of NCs as bioimaging agents, requiring stability, biocompatibility, deep-tissue penetration, and precise signal detection within living organisms. While early studies largely focused on visible and NIR-I emitting NCs (750–1000 nm), recent efforts have shifted toward developing probes that operate in the second near-infrared window (NIR-II, 1000–1700 nm). This spectral range offers clear advantages for biomedical imaging, including reduced tissue scattering, lower autofluorescence, and deeper penetration depths. These features collectively enhance spatial resolution and signal-to-background ratios, making NIR-II NCs particularly attractive for non-invasive diagnostics and image-guided interventions.

One of the pioneering approaches was reported by Song et al., who introduced cyclodextrin-protected AuNCs (CD-AuNCs) for antibody-guided NIR-II imaging of MCF-7 breast tumors [46]. With a compact core size of 1.85 nm and emission centered at 1050 nm, these NCs leveraged host–guest supramolecular chemistry for conjugation with anti-CD326 antibodies. The resulting Ab@AuNCs achieved ~12% injected dose (ID) tumor accumulation, approximately 4× higher than untargeted controls, and provided a threefold increase in imaging contrast at tissue depths up to 9 mm, outperforming conventional NIR-I agents. Notably, these ultrasmall NCs exhibited rapid renal clearance (>75% ID in 24 h) with no off-target toxicity, setting a benchmark for clinical translation. Adding subcellular precision to tumor imaging, Nie et al. engineered DNA-aptamer stabilized AuNCs with programmable valence to direct intracellular distribution in 4T1 breast cancer models [47]. These ultrasmall clusters emitted at 1030 nm and were functionalized with 1–4 AS1411 aptamers, controlling uptake into nuclei (V1–V2) or membrane retention (V4). Tumor accumulation increased with valence (up to 2.88% ID/g), and clearance remained efficient. The study introduced a unique strategy to fine-tune NC by discrete valence engineering without the need for post-synthetic modification.

**Figure 7:**
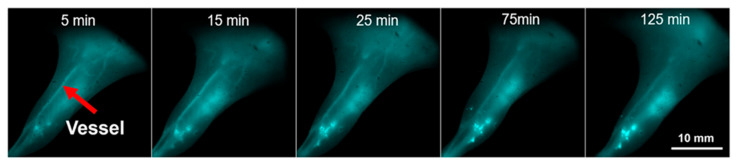
NIR-II dynamic imaging of leg vasculature using Au_7_Cd_1_-MHA/MPA NCs. Reprinted with permission from Ref. [49]. Copyright 2023 American Chemical Society.

Beyond oncology, Zhao et al. introduced tri-phe-nylphosphine-3,3′,3″-trisulfonic acid-capped ultrasmall AuNCs (TPPTS-AuNCs) for non-invasive detection of early-stage kidney injury [48]. These clusters remained non-emissive until activated by intracellular GSH via in situ ligand exchange, triggering NIR-II emission at ~1026 nm. This biomarker-free activation strategy produced high imaging contrast in inflamed renal tissue with minimal background signal and clear accumulation in renal tubular epithelium. Imaging lasted > 6 h post-injection and significantly outperformed always-on analogs, offering a new platform for activatable disease diagnostics. For high-resolution vascular imaging, Guo et al. synthesized Cd-doped dual-ligand Au7Cd1 NCs using 6-mercaptohexanoic acid (MHA) and 3- mercaptopropionic acid (MPA) stabilization [49]. These bright, photostable emitted in the NIR-II window (~1050 nm) and showed strong renal clearance, peaking in the bladder at ~90 min. Using InGaAs-based NIR-II fluorescence imaging, they visualized cerebral, hindlimb, and spinal vasculature in C57BL/6 mice, resolving vessels as small as 0.4 mm (Figure 7) with a high signal-to-noise ratio (as high as 11).

Their stability in serum and long imaging window (>270 min) highlight their suitability for real-time angiographic monitoring. Finally, Liu et al. explored the utility of Au25(SG)18 NCs in 3D volumetric imaging using Airy beam-assisted NIR-II light-sheet microscopy [50]. These glutathione-stabilized clusters emitted in both NIR-I and NIR-II windows (850–950 nm and 1150–1400 nm, respectively) and were employed to visualize ex vivo brain, thymus, spleen, and intestines in radiation-injured mice. Imaging with an 808 nm or 730 nm excitation source enabled deep-tissue penetration (~2.5 mm) and improved axial resolution (3.5 µm) across wide fields of view (~600 µm). This study demonstrated that AuNCs can serve as effective volumetric imaging agents for mapping radiation-induced damage, with minimal photobleaching and enhanced contrast using Airy beam illumination.

Together, these studies highlight the remarkable versatility of NIR-II-emitting AuNCs across diverse in vivo imaging applications, from tumor targeting and subcellular localization to kidney diagnostics, vascular mapping, and deep-tissue volumetric imaging. Their tunable surface chemistry, compact size, and high renal clearance efficiency position them as strong candidates for safe, high-contrast bioimaging. Moreover, the ability to engineer emission activation, dual-modal readouts, and precise targeting strategies underscores their adaptability for complex biological environments.

Overall, the studies discussed in the fluorescence imaging section illustrate the versatility of metallic NCs as imaging probes across in vitro, ex vivo, and in vivo models, highlighting their tunable emission, biocompatibility, and capacity for targeted visualization. To consolidate these findings and provide a comparative perspective, Table 2 summarizes representative examples of NCs employed for fluorescence imaging in different biological settings.

Despite their remarkable potential, metal NCs still face important limitations that must be addressed before widespread translation into biomedical imaging and theranostic applications. One of the main challenges is synthetic reproducibility, as small variations in reaction conditions can yield heterogeneous mixtures that complicate mechanistic interpretation and hinder regulatory approval. PL quantum yields, though improved in recent reports, often remain lower than those of established fluorophores, limiting sensitivity in deep-tissue imaging. In biological environments, stability and aggregation are persistent issues, particularly under high ionic strength or in the presence of competing biomolecules. Concerns about long-term toxicity, immunogenicity, and clearance pathways also remain insufficiently understood, especially for clusters that do not follow renal clearance routes. From an application standpoint, the limited penetration depth of NIR-I probes restricts their utility in clinical imaging, while NIR-II NCs, though promising, are still at an early developmental stage with few systematic toxicity and pharmacokinetic studies. To overcome these challenges, future efforts should focus on standardized synthesis and purification protocols to ensure batch-to-batch reproducibility, surface engineering with biocompatible and responsive ligands to improve stability and targeting, and integration with multimodal imaging or theranostic platforms to maximize functional versatility. Additionally, the incorporation of advanced spectroscopic methods for mechanistic studies will be crucial to link structure, dynamics, and function, ultimately guiding the rational design of next-generation NC probes. New ligand classes that have not yet been systematically applied, such as zwitterionic short-chain thiols for antifouling, N-heterocyclic carbenes for improved photostability, or glycan-derived thiols for receptor-specific uptake, could further expand the functional scope of NCs in bioimaging. From a commercialization perspective, the absence of standardized large-scale production methods, insufficient toxicological data, and the lack of regulatory approval pathways tailored to atomically precise nanomaterials currently limit clinical adoption. Overcoming these barriers will require collaborative efforts between researchers, industry, and regulatory agencies to establish good manufacturing practices, perform comprehensive long-term safety evaluations, and define regulatory frameworks. Expanding toward hybrid ligand shells that combine antifouling stability with disease-responsive or targeting moieties could represent a new direction to bridge laboratory demonstrations and clinical deployment.

### 3.2. Theranostics

While high-resolution NIR imaging provides valuable insight into disease at the molecular and tissue level, the next logical step is to combine diagnostic precision with therapeutic intervention. This combined strategy, known as theranostics, uses metal NCs not only as imaging probes but also as platforms for targeted therapy. By linking real-time visualization with treatment delivery, NC-based theranostics allow spatially and temporally precise therapies, minimize damage to healthy tissues, and enable dynamic monitoring of treatment response [38]. Recent research has focused on integrating NIR imaging with photothermal therapy (light-induced heating), photodynamic therapy (light-activated generation of reactive oxygen species), radiotherapy sensitization, or drug delivery, positioning NCs as promising tools for personalized medicine.

**Figure 8:**
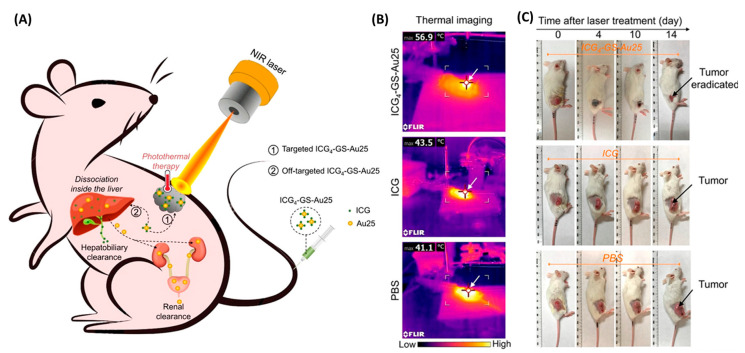
(**A**) Schematic illustration of the photothermal therapy approach for tumor targeting and cancer treatment using ICG_4_-GS-Au_25_ NCs under 808 nm laser irradiation. (**B**) Representative thermal images of mice subjected to photothermal therapy after 8 min of laser irradiation. White arrows highlight the tumor regions. The temperature in the top-left temperature of each image reprents the maximum temperature recorded in the measured area. (**C**) Representative color photographs of the tumors at different time points following photothermal therapy treatment. Reprinted with permission from Ref. [51] with permission from American Chemical Society, Copyright 2020.

One landmark example comes from Jiang et al., who developed indocyanine green (ICG)-conjugated GSH-stabilized Au_25_ NCs (ICG_4_–GSH–Au_25_) for breast cancer therapy [51]. Each ultrasmall Au_25_ cluster was conjugated with four ICG molecules. In their native state, fluorescence was quenched, but inside cells, thiol-triggered release restored emission, enabling imaging of tumor uptake and liver processing. Upon irradiation with an 808 nm laser, the construct produced localized heating (~55 °C) and completely ablated tumors, with high photothermal efficiency and minimal systemic toxicity (Figure 8).

Importantly, the clearance followed two routes, renal for AuNCs and hepatobiliary for ICG, helping to address long-term biocompatibility concerns. Kong et al. developed a cost-effective alternative by loading the dye Neutral Red (NR) into Min-23 peptide–stabilized AuNCs, creating NR@Min-23@AuNCs for breast cancer therapy [52]. These NCs emitted in the NIR-II region (~1050 nm), accumulated passively in tumors through the enhanced permeability and retention effect, and generated reactive oxygen species upon low-power light activation (8 mW/cm^2^). The result was ~90% tumor inhibition. With high renal clearance (~80% within 48 h) and simple activation, this platform shows potential even in low-resource clinical settings. Moving beyond phototherapy, Wang et al. designed AuNC–PTEN complexes for gene therapy in liver cancer [53]. PTEN (phosphatase and tensin homolog) is a tumor suppressor gene often lost in hepatocellular carcinoma. By co-administering Au precursors and PTEN DNA, the NCs self-assembled at the tumor site under reductive conditions. This allowed simultaneous tumor imaging and gene delivery. Compared to free PTEN or NCs alone, the AuNC–PTEN complex more effectively suppressed tumor growth, with minimal accumulation in healthy tissues. For inflammatory disease, Yang et al. introduced Au_44_ clusters stabilized by 4-mercaptobenzoic acid (Au_44_MBA_26_) for rheumatoid arthritis (RA) therapy [54]. These clusters targeted bone tissue by binding phosphate groups in hydroxyapatite, enabling NIR-II imaging (1080/1280 nm) and anti-inflammatory treatment in arthritic rats. They suppressed key inflammatory cytokines (TNF-α, IL-6, IL-1β), modulated the NF-κB pathway, and outperformed methotrexate in reducing joint inflammation and restoring bone integrity.

The versatility of NCs is further demonstrated by AgNCs coated with polyethylene glycol (PEG) and hyaluronic acid (HA) [55]. PEG provided stability, while HA targeted CD44 receptors, which are overexpressed on cancer cells. In breast tumor mouse models, these Ag@PEG2000-HA NCs produced both NIR emission (620–800 nm) for imaging and therapeutic effects by releasing Ag ions that disrupted mitochondrial function. The system inhibited tumor angiogenesis, metastasis, and proliferation, with high tumor selectivity and minimal off-target toxicity. In a complementary strategy, Zhao et al. developed Au–gadolinium (Au–Gd) NCs for combined NIR-II fluorescence imaging and magnetic resonance imaging (MRI), while also serving as radiosensitizers [56]. Prepared via albumin-mediated synthesis and functionalized with diethylenetriaminepentaacetic acid (DTPA)–Gd^3+^, these NCs showed strong NIR-II emission and exceptionally high MRI relaxivity (22.6 s^−1^·mM^−1^), surpassing clinical Gd agents. In glioma-bearing mice, they provided precise tumor localization and, when combined with radiotherapy (8 Gy X-ray), tripled survival and significantly reduced tumor burden. Their dual clearance pathways (renal and hepatic) and favorable biocompatibility make them promising candidates for image-guided radiotherapy.

Finally, theoretical work by Abd El-Mageed et al. explored AuNCs as nanocarriers for the anticancer drug D-penicillamine (DPA) using computational modeling [57]. The study demonstrated stable binding via covalent and hydrogen-bond interactions, along with feasible drug release in aqueous media. While preliminary, this highlights the potential of atomically precise NCs as customizable drug carriers. Together, these studies show the broad potential of NCs as next-generation theranostic agents. Their tunable size, surface chemistry, and optical properties, especially in the NIR region, enable precise integration of imaging and therapy across a wide range of diseases, from solid tumors to inflammatory disorders. Depending on the design, NCs can achieve photothermal tumor ablation, light-triggered photodynamic therapy, targeted gene delivery, anti-inflammatory modulation, or multimodal imaging with radiotherapy sensitization. Importantly, these therapeutic actions are consistently achieved with low systemic toxicity and efficient clearance. To provide a consolidated overview, Table 3 summarizes representative NC-based theranostic systems.

However, while these examples illustrate the versatility of NCs as theranostic platforms, several limitations still need to be addressed before clinical translation. First, photothermal and photodynamic efficiencies are often lower compared to NP-based agents, and improved strategies to enhance quantum yields and energy conversion are required. Second, synthetic reproducibility and large-scale production remain challenging, with batch-to-batch variability affecting both therapeutic and imaging performance. Third, although many reports emphasize rapid renal clearance, systematic long-term toxicity, biodistribution, and immunogenicity studies are still scarce and must be performed across multiple animal models. Furthermore, most current studies rely on proof-of-concept cancer models, and broader evaluation in complex or chronic diseases is necessary to fully establish the translational potential of NC-based theranostics. To overcome these limitations, future work should focus on the standardization of synthesis and purification protocols, rational surface engineering with multifunctional ligands to improve stability and therapeutic payload delivery, and the integration of multimodal imaging techniques, such as NIR-II microscopy combined with MRI or photoacoustic imaging, to maximize diagnostic precision. New ligand classes that remain underexplored in theranostics include enzyme-cleavable peptides that release therapeutic cargo specifically in tumor sites, porphyrin- or BODIPY-functionalized ligands to directly enhance photodynamic efficiency, and glycan-derived ligands to target metabolic or immune pathways beyond oncology. From a commercialization perspective, the barriers are closely aligned with those identified for imaging applications: the absence of scalable manufacturing processes, incomplete toxicological and pharmacokinetic profiling, and the lack of clear regulatory frameworks tailored to atomically precise nanomaterials. Addressing these challenges through the establishment of good manufacturing practices, comprehensive long-term safety assessments, and collaboration with regulatory agencies will be essential. With such efforts, NC-based theranostic systems could progress from experimental demonstrations to clinically viable technologies in the near future.

### 3.3. Sensing

The unique physicochemical properties of metal NCs make them highly attractive also for chemical and biological sensing. Their discrete energy states allow for bright photoluminescence, while their surface chemistry can be engineered to selectively interact with a wide range of analytes. As a result, NCs have emerged as versatile probes capable of converting molecular recognition events into measurable optical signals [58,59]. Among the most explored applications is the detection of ions, where NCs serve as both recognition elements and signal transducers. Their fluorescence is often modulated through direct coordination, redox interactions, or aggregation effects upon ion binding, enabling sensitive and selective quantification of biologically and environmentally relevant species. Beyond ions, NCs have also proven effective for sensing small molecules by leveraging tailored surface ligands or catalytic activities that induce measurable changes in their optical response. In recent years, attention has expanded toward pathogen detection, where NCs are increasingly used in combination with biomolecular recognition elements such as aptamers, antibodies, or nucleic acid sequences. These platforms exploit the signal amplification potential and biocompatibility of NCs to achieve high sensitivity in detecting bacteria, viruses, or pathogen-derived markers. In the following section, we will explore representative sensing applications of NCs, beginning with ion detection, then small molecules, and finally pathogen-related targets, highlighting both the detection mechanisms and innovations that contribute to their selectivity, sensitivity, and real-world applicability.

#### 3.3.1. Ion Detection

Metal NCs have emerged as powerful tools for ultrasensitive ion detection, owing to their tunable fluorescence and ease of functionalization. Their ability to detect metal ions through photoluminescence quenching or enhancement mechanisms makes them ideal for environmental monitoring and biomedical diagnostics. In this section, we present recent advances in NC-based ion sensors, beginning with systems targeting a single analyte, moving to multiplexed platforms, and concluding with innovative paper-based sensors suitable for naked-eye or smartphone-assisted detection.

Among single-ion detection systems, Zhao et al. developed GSH-stabilized AuNCs (GSH-AuNCs) emitting at 500 nm for selective cobalt detection [60]. The key innovation lies in pH-tuned fluorescence quenching: at pH 6.0, only Co^2+^ disrupts the Au–S interface via static quenching, enabling high specificity even in the presence of Cu^2+^ or other interfering ions. The sensor showed a linear range of 2.0–50.0 µM with a detection limit of 0.124 µM and was successfully validated on environmental water samples with recovery between 102.8% and 108.3%. Worth pointing out that in this review, the term recovery refers specifically to the recovery rate in spiked sample analyses, the percentage of analyte detected relative to the amount initially added. This value may occasionally exceed 100% due to experimental or matrix effects. Furthermore, beyond accuracy, the GSH-AuNCs-based sensing platform also demonstrated high reproducibility (relative standard deviations (RSD) < 0.4%) and a visible fluorescence response under UV light. A different ion-specific system was proposed by Singh et al., who fabricated BSA-stabilized CuNCs for Fe^3+^ detection [61]. The probe, emitting blue fluorescence at 405 nm, relies on static quenching and inner filter effect (IFE) upon Fe^3+^ binding, without significant lifetime changes. It achieved a low LOD of 10 nM over a 0.2–2.4 µM range and was tested in natural water, wastewater, and human serum, with recoveries of 93–104% and RSDs below 5.8%. The sensor’s high sensitivity and biocompatibility make it promising for clinical and environmental use. Furthermore, Zhang et al. introduced a thiol-functionalized CuNC probe for Ag^+^ sensing based on aggregation-induced quenching [62]. These MMI-capped CuNCs emitted at 476 nm with a lifetime of 10.57 µs, and upon Ag^+^ binding, formed aggregates that reduced fluorescence through static quenching. The sensor achieved a LOD of 6.7 nM within a 0.025–50 µM range and exhibited excellent selectivity against numerous metal ions. Tested in human blood serum, the sensor delivered recoveries of 97–104% (RSD < 3.6%). The authors also demonstrated the material’s multifunctionality for anticounterfeiting and LED applications, showcasing its high stability and practical versatility.

In terms of multi-ion detection, Desai et al. developed AuNCs from Curcuma longa extract capable of independently sensing Cd^2+^, Zn^2+^, and Cu^2+^ ions [63]. The green-emitting AuNCs (emission at 619 nm) showed distinct detection mechanisms: fluorescence enhancement for Cd^2+^/Zn^2+^ and fluorescence quenching for Cu^2+^. They achieved nanomolar sensitivity (LOD: Cd^2+^ = 12 nM, Zn^2+^ = 16 nM, Cu^2+^ = 26 nM), with linear ranges of 10 nM–10 µM. Tested in biological and various digested food samples, they showed recovery between 87 and 103%. Another multi-ion detection platform was reported by Saleh et al., who used coffee extract-mediated AuNCs for Cu^2+^ and Hg^2+^ detection [64]. These bright green-emitting NCs (507 nm emission) relied on different quenching pathways: Cu^2+^ induced chelation with functional groups, while Hg^2+^ interacted with surface Au^+^. Detection was made selective through masking agents: NaBH_4_ for Hg^2+^ and EDTA for Cu^2+^. The system achieved LODs of 14.78 nM for Cu^2+^ and 35.21 nM for Hg^2+^, with excellent selectivity and reproducibility over four cycles. Applied to tap water, both ions were accurately detected with recoveries above 96% and RSD < 1.6%.

**Figure 9:**
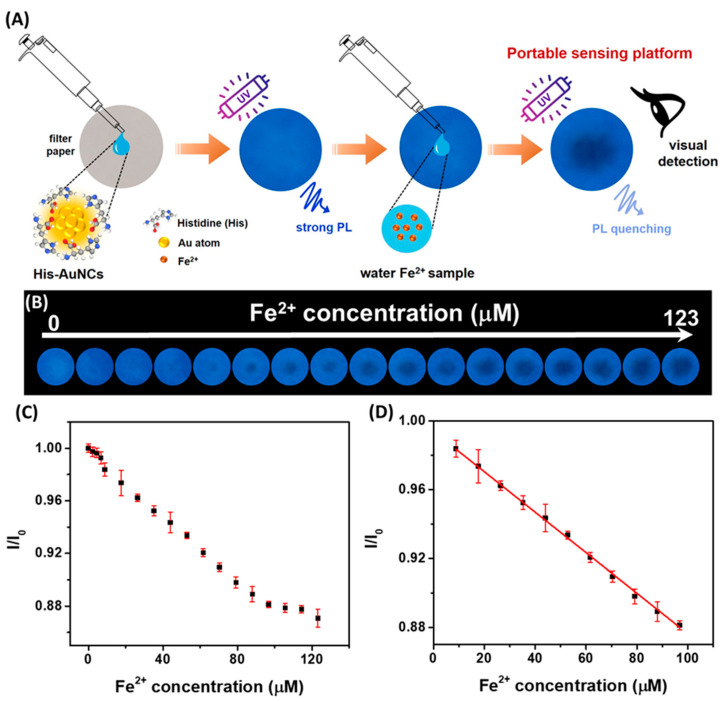
(**A**) Schematic illustration of Fe ion detection using a paper-based sensing platform with incorporated histidine-stabilized AuNCs. (**B**) Photographic images of histidine-stabilized Au NCs (His-AuNCs) deposited on paper, recorded under UV excitation 15 min after exposure to solutions containing different concentrations of Fe^2+^ (0–123 µM). (**C**) Variation in the average blue intensity ratio (I/I_0_) of His-AuNCs-paper spots as a function of Fe^2+^ concentration in the 0–123 µM range. (**D**) Corresponding calibration plot with linear fitting, demonstrating a linear dynamic range between 9 and 97 µM. Reprinted with permission from Ref. [19]. Copyright 2022 Multidisciplinary Digital Publishing Institute.

Among the most accessible and user-friendly platforms for ion sensing are paper-based NC sensors, which enable visual detection and smartphone-assisted quantification without the need for specialized equipment. A practical approach first explored by our group employed BSA-stabilized AuNCs (BSA-AuNCs) emitting at 670 nm for the detection of Cu^2+^ ions [65]. In this format, photoluminescence was quenched through Cu^2+^ chelation with BSA cysteine residues. The system supported both solution-based quantification, offering a dual linear range (0–17 µM and 17–1724 µM) and a low detection limit of 0.83 µM, and paper-based sensing that enabled naked-eye detection under UV light down to 5 µM. The paper strips remained functional for at least 14 days and performed reliably across a variety of water samples, illustrating the potential for low-cost, eco-friendly field diagnostics. Building on this platform, our group later developed a more advanced paper-based sensing system for iron ion detection using histidine-stabilized AuNCs (His-AuNCs) synthesized via a microwave-assisted method (Figure 9A) [19].

These blue-emitting NCs (471 nm) enabled detection of both Fe^2+^ and Fe^3+^ ions, with application in both solution and paper formats. The paper-based strips supported rapid visual detection under UV light, while the integration of smartphone image analysis allowed for precise quantification, achieving a detection limit of 3.2 µM (Figure 9B–D). When tested on real water sources, including river, spring, and tap water, the sensor delivered recoveries between 102% and 105.4%, highlighting its potential for portable, accessible environmental monitoring.

Metal NCs have proven to be highly effective platforms for ion detection, achieving excellent sensitivity, selectivity, and stability across both single-ion and multi-ion sensing formats. Their successful application in real samples, including environmental waters and biological fluids, highlights their practical relevance. Notably, paper-based NC sensors bring added value through portability, low cost, and ease of visual or smartphone-assisted readout. Looking ahead, the integration of NC-based sensors into smart, low-power devices, such as smartphone-linked diagnostics and wearable formats, will likely drive widespread adoption in environmental surveillance and point-of-care testing. Continued innovation in ligand chemistry, doping strategies, and surface engineering will enable enhanced selectivity, stability, and multiplexing capabilities. Furthermore, bridging sensing with real-time data transmission and AI-based analysis could unlock automated contaminant tracking and health monitoring systems. As regulatory pressures grow for on-site and decentralized monitoring, NC-based sensors are poised to become essential components of next-generation analytical technologies.

#### 3.3.2. Small Molecules Detection

The application of metal NCs in sensing small molecules has expanded significantly, with tailored platforms now offering high sensitivity, selectivity, and practical deployment across diverse analyte classes. Among the earliest examples targeting pesticides, Yang et al. developed a ratiometric fluorescence assay for carbendazim (CBZ) detection using a nanohybrid of N-doped carbon quantum dots and AuNCs [66]. In this dual-signal platform, the addition of CBZ disrupts FRET-induced quenching, leading to fluorescence recovery and enhanced Rayleigh scattering. The assay showed broad dynamic ranges (1–100 µM and 150–1000 µM), a LOD of 0.83 µM, and excellent recovery in spiked fruit samples, providing a rapid (~10 min), label-free solution with high selectivity. Complementarily, Yixia Yang et al. targeted glyphosate, employing a Cu^2+^-modulated DNA-templated AgNCs (DNA-AgNCs) sensor [67]. Glyphosate chelated Cu^2+^ ions that otherwise quenched the DNA-AgNCs, triggering a fluorescence turn-on effect. The sensor achieved a LOD of 5 µg/L and excellent selectivity over similar organophosphorus pesticides. It was successfully validated in mineral and tap water, offering a cost-effective tool for environmental glyphosate monitoring.

For biomolecular detection, several strategies highlight the versatility of NCs. Zhou et al. designed a DNA-AuNC-based platform for sensing DNA methyltransferase (MTase) activity, relevant to epigenetic cancer diagnostics [68]. MTase-mediated cleavage and extension of DNA transformed the NC environment, quenching fluorescence via PET from G-rich sequences. The system demonstrated a LOD of 0.178 U/mL and retained high accuracy in human serum and cancer cell lysates. Notably, it also enabled inhibitor screening for 5-Azacytidine, illustrating its dual diagnostic and drug discovery potential. For multiplex detection, Qin et al. anchored AuNCs onto Fe_3_O_4_@SiO_2_ magnetic nanocomposites using a novel tris(2-carboxyethyl)phosphine (TCEP)-mediated immobilization method [69]. With duplex-specific nuclease-assisted amplification, simultaneous detection of miRNA-21 and miRNA-141 was achieved, reaching femtomolar LODs (0.020 and 0.017 pM, respectively) and excellent specificity in serum and urine. This dual-analyte detection platform demonstrated magnetic enrichment, precise selectivity, and signal amplification, offering a powerful biosensing tool for clinical miRNA profiling. In the context of antioxidant monitoring, Qi et al. employed sanguinarine-templated CuNCs for detecting ascorbic acid (AA) [70]. Here, MnO_2_NS quenched CuNC fluorescence via aggregation, while the presence of AA resulted in MnO_2_NS degradation and restored signal, producing a sensitive “turn-on” mechanism with a LOD of 6.9 µM. The method proved applicable to orange drinks and vitamin tablets, with recoveries of 94–105%. Finally, Bin Jardan et al. reported a PEI/DTH-stabilized nickel NCs platform for GSH detection [71]. Initially quenched by Fe^3+^ ions, fluorescence was restored by GSH, displacing Fe^3+^ via chelation. This system achieved an ultra-low LOD of 7.0 nM, rapid response (3 min), and high recovery in serum, urine, saliva, and supplements, demonstrating broad bioanalytical utility.

**Figure 10:**
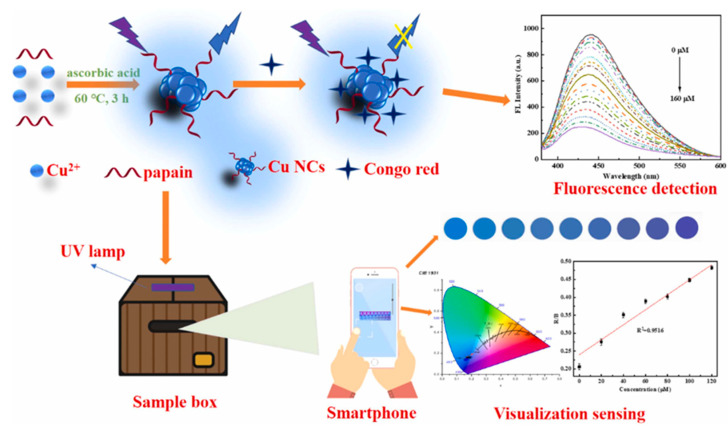
Schematic illustration of the sensitive and visual detection of Congo red using the CuNCs@PP fluorescence sensor. Reprinted with permission from Ref. [72]. Copyright 2025 Elsevier.

Shifting toward dyes, Zhang et al. reported a papain-stabilized CuNC (CuNC@PP) platform for Congo red detection [72]. The probe relied on the inner filter effect (IFE), wherein the dye absorbed excitation and emission wavelengths, causing fluorescence quenching. The system offered a linear range of 0.5–160 µM with a low LOD of 0.085 µM via fluorescence, and 3.59 µM using smartphone RGB analysis (Figure 10). High salt, pH, and photostability, along with successful application in river and tap water, highlight its field-readiness. The smartphone integration also added value for on-site, low-cost dye contamination monitoring.

The use of metal NCs in small molecule sensing has advanced considerably in recent years. From ratiometric and turn-on fluorescence platforms for pesticide and antioxidant detection to DNA-guided and magnetic nanocomposite-based biosensors for disease biomarkers, NCs have shown excellent sensitivity, selectivity, and robustness. Their modularity has enabled fine-tuned signal transduction mechanisms, including FRET modulation, PET-based quenching, and coordination-triggered recovery, yielding low detection limits and reliable performance in real-world samples such as fruit extracts, beverages, water, serum, and urine. Future efforts in small molecule sensing with NCs will likely emphasize integration into portable, miniaturized devices. Innovations in ligand design, ratiometric dual-emission systems, and enzyme-free amplification strategies will enhance performance while maintaining simplicity and low cost. Additionally, expanding the repertoire of analytes, particularly clinically relevant metabolites and biomarkers, will drive translation into diagnostic and therapeutic monitoring applications. The continued development of multiplexed detection schemes and real-time monitoring capabilities will position NC-based sensors as valuable tools in next-generation point-of-need analytical systems.

#### 3.3.3. Pathogen Detection

Recent advances in pathogen detection have harnessed the unique optical and catalytic properties of metal NCs for the development of sensitive, selective, and field-deployable biosensing platforms. For bacterial detection, Evstigneeva et al. employed GSH-capped AuNCs (GSH–AuNCs), emitting at 612 nm, for the fluorescence imaging of Staphylococcus aureus and Escherichia coli biofilms [73]. These NCs selectively bound to the extracellular polymeric substances (EPS) within the biofilm matrix rather than penetrating bacterial cytoplasm, allowing for clear visualization using confocal laser scanning microscopy. The method achieved a detection limit of 1.7 × 10^5^ CFU/mL, showing a 10-fold improvement over traditional crystal violet staining assays, and maintained excellent photostability and low cytotoxicity, demonstrating its potential for non-invasive biofilm diagnostics. In the context of foodborne bacterial pathogens, Song et al. developed a dual-functional biosensor combining the peroxidase-mimicking activity of papain-stabilized AuNCs (papain@AuNCs) with the specificity of aptamers for *E. coli* O157:H7 [74]. Upon binding to the bacterial surface, the catalytic efficiency of the NCs toward TMB oxidation (in the presence of H_2_O_2_) was significantly enhanced, producing a quantifiable colorimetric signal at 652 nm visible to the naked eye. The sensor achieved an exceptional limit of detection of 39 CFU·mL^−1^ in pure cultures, and retained high sensitivity in ultra-high temperature (UHT), pasteurized, and raw milk (LODs ~500 CFU·mL^−1^), while showing negligible cross-reactivity against 16 other foodborne bacteria. This work marks one of the first demonstrations of aptamer-enhanced nanozyme activity of AuNCs for real-world dairy safety monitoring. In a similar direction, Pang et al. introduced a colorimetric biosensor targeting Salmonella typhimurium using aptamer recognition to trigger the release of complementary DNAs that assemble into a three-way junction (3WJ) DNA structure [75]. This template directed the in situ synthesis of Ag/Pt bimetallic NCs, which exhibited enhanced peroxidase-like activity. The system catalyzed the oxidation of 3,3′,5,5′-Tetramethylbenzidine (TMB) to generate a yellow signal at 450 nm upon acidification. The sensor achieved a LOD of 2.6 × 10^2^ CFU/mL in buffer and 7.2 × 10^2^ CFU/mL in commercial milk, and exhibited excellent specificity against multiple common pathogens. This strategy highlights the first application of 3WJ DNA scaffolds for NC templating and introduces a robust enzyme-free signal amplification approach adaptable to various targets.

For viral pathogen detection, Liu et al. reported a highly sensitive electrochemiluminescence (ECL) biosensor for Human Papillomavirus type 16 (HPV-16) DNA, integrating CRISPR/Cas12a with L-methionine-stabilized AuNCs (Met-AuNCs) [76]. The presence of HPV-16 DNA activated the Cas12a/crRNA complex, which cleaved ferrocene-labeled single-stranded DNA (SH-ssDNA-Fc), thereby restoring the quenched ECL emission from the Met-AuNCs. This “turn-on” response enabled quantification down to 0.48 pM over a 1 pM–10 nM range. Notably, the assay functioned in undiluted human blood with >95% recovery, confirming its translational potential for point-of-care diagnostics. This study was the first to demonstrate Met-AuNCs as ECL emitters in a CRISPR-based format. Extending this CRISPR strategy, Tao et al. constructed a fluorescence sensor for Hepatitis B Virus (HBV) DNA by exploiting Cas12a-mediated cleavage of single-stranded DNA, which inhibited the formation of fluorescent metal NCs [77]. Among AuNCs, AgNCs, and CuNCs evaluated, DNA-templated CuNCs outperformed in speed, photostability, and sensitivity, yielding a LOD of 0.54 pM within 25 min. The probe achieved >99% recovery in spiked serum and showed negligible cross-reactivity with hepatitis A virus, hepatitis C virus, and human immunodeficiency virus. Importantly, this platform circumvented the need for fluorophore–quencher labeling, offering a low-cost and label-free alternative for clinical virology.

For enzymatic biomarkers, Wu et al. presented a fluorescence “turn-off” biosensor for trypsin, utilizing protamine-enhanced fluorescence of polyadenine DNA-templated AuNCs [78]. Trypsin hydrolyzed protamine, destabilizing the DNA–AuNC complex and reducing its fluorescence at 475 nm. The sensor operated with a LOD of 1.5 ng/mL and maintained specificity against a range of non-target enzymes. When tested in diluted serum, it demonstrated recovery between 98.7 and 103.5%, suggesting high clinical relevance for detecting pancreatic function or related disorders. Finally, for the detection of food toxins, Niu et al. engineered a fluorescence aptasensor for Aflatoxin B1 (AFB1), combining catalytic hairpin assembly (CHA) with DNA nanoflower (DNF)-templated AuNCs and Mn-Metal–Organic Framework (Mn-MOF) spatial confinement [79]. The resulting DNF@AuNCs emitted at 442 nm, and signal quenching occurred through G-rich sequence binding in the presence of AFB1. This multi-layered amplification strategy yielded an ultrasensitive LOD of 7 pg/mL across 0.01–200 ng/mL, and the sensor performed robustly in spiked peanut and corn flours, with recoveries exceeding 95%. This work is notable for integrating in situ NC synthesis with CHA amplification and MOF confinement to enhance both specificity and signal strength.

Recent advances in NC-based pathogen detection highlight their versatility in tackling a broad spectrum of targets, from bacterial biofilms and foodborne pathogens to viral DNA and enzymatic biomarkers. These platforms exploit the photoluminescent, catalytic, and surface-tunable nature of NCs, enabling highly sensitive, selective, and often label-free assays. NCs have demonstrated excellent performance across complex biological and food matrices, offering both visual and quantitative readouts. Their integration with aptamers, DNA scaffolds, and bio-recognition elements has enabled specific target recognition with low limits of detection, while maintaining biocompatibility and rapid response times. Looking ahead, pathogen detection using NCs is expected to further evolve toward portable, field-deployable platforms combining smartphone-based quantification, paper-based formats, and low-cost synthesis. The incorporation of programmable recognition systems like CRISPR/Cas and aptamer logic gates will facilitate multiplexed detection and dynamic response tuning. Additionally, expanding the role of NCs in enzyme-free catalytic amplification, combined with advances in material scaffolding (e.g., MOFs, DNA origami), will allow for higher signal gain and broader applicability. Future directions should focus on clinical validation, regulatory approval, and integration into point-of-care diagnostics and food safety surveillance, bringing NC-based sensing technologies closer to real-world deployment.

As discussed, metallic NCs provide versatile sensing platforms capable of detecting ions, small molecules, and pathogens with high sensitivity and selectivity. Their tunable surface chemistry and responsive photoluminescence make them particularly effective in translating molecular recognition into measurable optical signals across diverse environments. To consolidate these advances, Table 4 summarizes representative examples of NCs employed in sensing applications.

Notwithstanding these advances, several factors still constrain the practical deployment of NC-based sensors. Matrix effects and environmental interferents can bias quantification (including apparent recoveries above 100%), while variability in synthesis and purification undermines batch-to-batch reproducibility. Operational stability also remains a concern: photobleaching, signal drift in complex media, and limited shelf-life complicate translation into portable formats. Moreover, most systems have not yet been validated systematically in real samples under standardized protocols or benchmarked against regulatory performance criteria. Progress will require harmonized, scalable syntheses with defined quality control, robust surface engineering to mitigate aggregation in complex matrices, and measurement strategies that reduce environmental bias, for example, ratiometric or lifetime-based readouts. Beyond the analyte-specific ligands, new classes that could enhance sensor robustness include zwitterionic antifouling thiols to suppress nonspecific adsorption in food and environmental matrices, short peptide-based stabilizers that provide controlled surface chemistry and long shelf-life, and polymeric anchors that are compatible with paper-based or microfluidic formats. From a commercialization standpoint, the most immediate opportunities lie in environmental monitoring and food-safety screening, particularly paper-based and smartphone-assisted platforms, where regulatory pathways are comparatively tractable and required biocompatibility burdens are lower. Here, advancement depends on establishing calibrated manufacturing, device-agnostic image or signal standardization, and field validation across diverse water and food matrices. By contrast, clinical diagnostics will demand comprehensive toxicological and biocompatibility assessments (for in vivo or invasive use), interlaboratory method concordance, and conformity with the requirements of in vitro diagnostic (IVD) regulation and Clinical Laboratory Improvement Amendments (CLIA) certification. Pairing NCs with orthogonal modalities (magnetic enrichment or electrochemiluminescence) and embedding assays in microfluidic devices can improve specificity, automation, and user independence. Looking forward, expanding NC-based sensors into non-traditional applications such as wearable health monitoring or on-site pathogen screening could open new avenues for translation beyond current environmental and food contexts.

### 3.4. Catalysis

Catalysis represents one of the most dynamic frontiers for metal NCs, enabling atom-efficient transformations that are central to energy, environmental, and synthetic chemistry [80]. With their precisely defined atomic arrangements, abundant low-coordinated surface atoms, and size-dependent electronic structures, NCs offer catalytic behaviors that differ fundamentally from larger NPs or bulk metals [81,82,83,84]. Their tunable reactivity, combined with ligand-directed surface modulation, makes them especially appealing for activating small molecules under mild conditions.

This section highlights recent advances in NC-driven catalysis, with a focus on key sustainable processes: CO_2_ reduction, water splitting for hydrogen evolution, dye degradation for wastewater remediation, N_2_ fixation, H_2_O_2_ generation. These systems not only demonstrate high activity and selectivity but also offer mechanistic insights into how atomic precision, support effects, and electronic modulation govern catalytic performance.

#### 3.4.1. CO_2_

**Figure 11:**
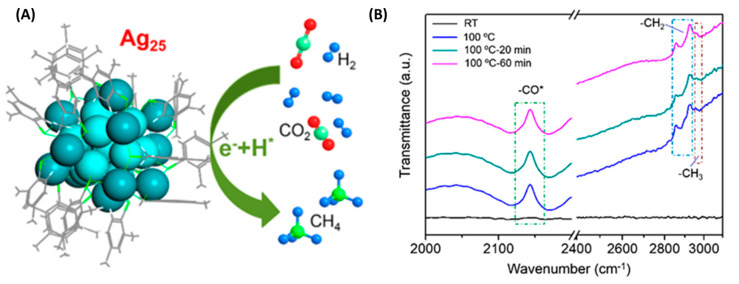
(**A**) Schematic illustration of the catalytic reaction of CO_2_ to CH4 in the presence of AgNCs under light irradiation. (**B**) Time-resolved operando IR spectra of reaction intermediates on Ag_25_ clusters at 100 °C for CO_2_. Reprinted with permission from Ref. [85] Copyright 2021 American Chemical Society.

The catalytic reduction of CO_2_ using atomically precise metal NCs has garnered significant attention due to their molecularly tunable active sites, well-defined structures, and efficient light-harvesting capabilities. In the context of methane (CH_4_) production, Xiong et al. demonstrated the use of Ag_25_(SPhMe_2_)_18_ NCs for photodriven CO_2_ hydrogenation (Figure 11) with exceptional performance [85]. These ~1.5 nm clusters feature an icosahedral Ag_13_ core and exhibited nearly 100% selectivity toward CH_4_, outperforming larger Ag nanoparticles, which showed no activity under identical conditions.

IR spectroscopy and DFT simulations revealed a multistep reduction pathway involving adsorbed CO_2_, conversion to formyl and formaldehyde intermediates, and surface CHₓ formation prior to methane evolution. The discrete molecular orbitals of Ag_25_, coupled with their long photoluminescence lifetime and strong light absorption, were critical in promoting multielectron transfer under visible light. When carbon monoxide (CO) is the desired product, Au-based NCs have also shown remarkable efficiency and selectivity. Jiang et al. developed a hybrid catalyst by immobilizing ultrasmall AuNCs (~1.8 nm) onto a UiO-68 Zr-based MOF functionalized with N-heterocyclic carbenes (NHCs) [86]. This covalently linked Au–NHC–MOF interface facilitated charge separation and efficient electron transfer from light-excited AuNCs into the MOF conduction band, enhancing CO_2_ activation. The resulting system achieved a CO evolution rate of 57.6 mmol·g^−1^·h^−1^, significantly surpassing the performance of physical Au/MOF mixtures, and showed stable operation over multiple cycles with minimal generation of H_2_ or CH_4_. A different design by Tian et al. used ligand-free Au_25_ NCs deposited on BiOBr nanosheets [87]. These clusters served both as charge acceptors and catalytic centers, improving interfacial charge transfer and lowering energy barriers for the formation of key intermediates like *COOH and *CO, as confirmed by DFT. The CO generation rate increased to 43.57 µmol·g^−1^·h^−1^, nearly 3-fold that of pristine BiOBr, with high selectivity for CO and excellent photocatalytic stability over 25 h.

Among Cu-based NC systems, several strategies have targeted either CO or formic acid (HCOOH) production. Dong et al. designed a crystalline Cu_6_–NH cluster coordinated by 2-mercaptopyrimidine ligands, where protonated nitrogen atoms act as intramolecular proton donors, effectively relaying protons to CO_2_ molecules adsorbed on Cu sites [88]. This structure enabled a CO evolution rate of 148.8 µmol·g^−1^·h^−1^, with nearly 100% selectivity toward CO, and long-term operational stability. DFT calculations and in situ spectroscopies confirmed that the presence of hydrogen bonds significantly reduced the energy barrier for the *COOH intermediate formation, the rate-determining step in CO_2_ reduction. Complementary work by Dai et al. introduced ultrasmall Cu NCs (~1.6 nm) embedded in Zr-based MOFs (MOF-801 and UiO-66-NH_2_) via a seed-mediated growth method [89]. These core–shell structures promoted synergistic host–guest interactions, leading to enhanced charge separation and CO_2_ adsorption. Particularly, Cu NCs@UiO-66-NH_2_ favored formic acid production with 86% selectivity, achieving HCOOH rates up to 128 µmol·h^−1^·g^−1^ under UV light. The amino-functionalized MOF improved visible light absorption and created a favorable microenvironment for CO_2_ activation at Cu^+^/Cu^0^ interfacial sites.

Expanding beyond gas-phase products, Qiao et al. reported a novel approach for CO_2_ fixation into cyclic organic molecules via the carboxylative cyclization of propargylamines into oxazolidinones [82]. This transformation was catalyzed by a Cu_6_-NH_2_ NC featuring dual Lewis acid–base functionality, where Cu^+^ centers activated the alkyne moiety and terminal –NH_2_ groups stabilized amine–CO_2_ intermediates through hydrogen bonding. The catalyst operated under ambient conditions (1 atm CO_2_, 30 °C) without cocatalysts or solvents, achieving up to 99% yield and turnover frequencies (TOFs) of 387 h^−1^, markedly higher than traditional homogeneous systems. Notably, it also demonstrated efficiency under simulated flue gas conditions, scalability to gram quantities, and reusability over five cycles with negligible loss of activity.

Therefore, atomically precise metal NCs exhibit high efficiency in catalyzing CO_2_ into high-value products such as methane, carbon monoxide, formic acid, and oxazolidinones, showcasing how their tunable active sites, discrete electronic structures, and strong light-harvesting properties enable selective multielectron transfer and efficient charge separation under mild conditions. Looking forward, future directions should focus on developing scalable and environmentally benign synthetic routes for metal NCs that retain atomic precision and structural integrity under catalytic conditions, integrating NC-based systems into photoelectrochemical devices for solar-to-fuel conversion, expanding the product scope beyond simple C_1_ compounds toward higher hydrocarbons or C–C coupled molecules, and ensuring catalyst robustness under realistic flue gas environments. Advancing mechanistic understanding through spectroscopies and computational modeling will be crucial for rational design, while the development of hybrid architectures combining NCs with porous or two-dimensional matrices holds great potential for enhancing activity, selectivity, and recyclability in practical CO_2_ valorization applications.

#### 3.4.2. Water Splitting

**Figure 12:**
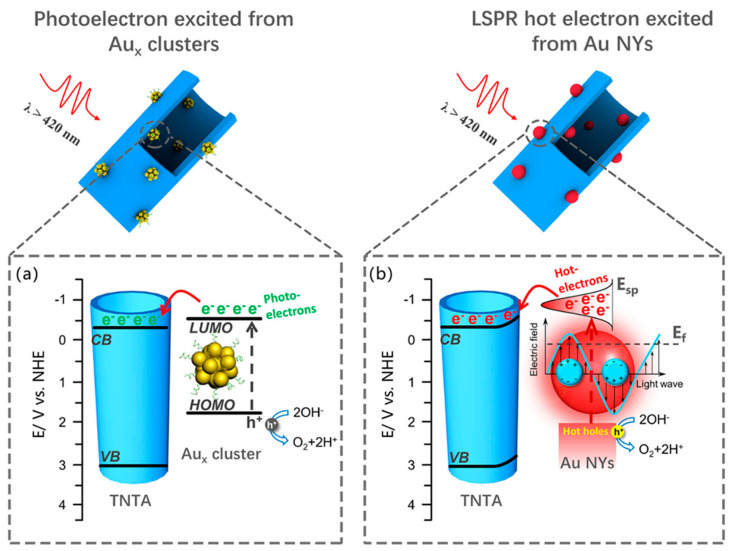
Schematic illustration depicting the charge transfer mechanism in (**a**) TNTAs-Aux and (**b**) plasmonic TNTAs-Au. Reprinted with permission from Ref. [92]. Copyright 2020 American Chemical Society.

The photocatalytic splitting of water into hydrogen and oxygen remains one of the most sought-after strategies for sustainable energy generation, and atomically precise metal NCs have emerged as versatile co-catalysts and active sites in this field due to their well-defined structures, tunable energy levels, and superior charge separation properties. One of the foundational examples is the work of Wang et al., who explored the integration of thiolate-protected Ag_44_(SR)_30_ NCs with commercial TiO_2_ NPs to form a type II heterojunction interface where the conduction band of TiO_2_ is lower in energy than the conduction band of the NCs, which dramatically enhanced hydrogen evolution under simulated sunlight [90]. The Ag_44_ NCs, with their five distinct absorption bands and narrow HOMO–LUMO gap of ~0.77 eV, functioned dually as photosensitizers and co-catalysts. Upon UV-Vis excitation, holes generated in TiO_2_ were effectively transferred to the HOMO of Ag_44_, while electrons from the excited Ag NCs were injected into the TiO_2_ conduction band, resulting in efficient charge separation and suppressed recombination. Ultrafast spectroscopy and transient absorption data revealed extended charge carrier lifetimes (>2 ns), which translated into a remarkable hydrogen evolution rate of 7.4 mmol·h^−1^·g^−1^, ten times higher than bare TiO_2_ and comparable to TiO_2_-Pt systems, without the use of noble metal NPs. In a complementary strategy, Fu et al. harnessed the inherent instability of GSH-capped AuNCs (Au_x_@GSH) as a functional asset rather than a limitation [91]. They constructed a complex heterostructure comprising CdS nanowires enveloped by CdTe and PDDA layers, onto which Au_x_@GSH NCs were deposited. Under visible light, these Au NCs underwent in situ transformation into small Au nanocrystals (~3 nm), which, rather than diminishing performance, acted as efficient electron sinks. The layered CdS@CdTe@PDDA@Au_x_ structure formed a cascade energy alignment that guided photoexcited electrons from CdS to the Au domains via CdTe and PDDA intermediates, greatly improving electron migration and interfacial redox kinetics. This architecture achieved a hydrogen evolution rate of 4.42 mmol·g^−1^·h^−1^, more than 14-fold greater than pristine CdS, with a solar-to-hydrogen (STH) efficiency of 24.1% and remarkable stability over multiple cycles. Another study by Dai et al. systematically compared the performance of glutathione-capped AuNCs versus larger plasmonic Au nanocrystals when deposited on TiO_2_ nanotube arrays (TNTAs) for photoelectrochemical (PEC) water splitting [92]. They showed that Aux@GSH clusters (including Au_25_(GSH)_18_) significantly outperformed their plasmonic counterparts in terms of photocurrent generation (Figure 12), charge carrier density, and applied bias photon-to-current efficiency (ABPE).

The TNTAs–Aux heterostructure generated a photocurrent density of 0.07 mA/cm^2^, compared to only 0.015 mA/cm^2^ for TNTAs–Au. This superiority was attributed to the molecular-like nature of AuNCs, which promoted direct LUMO-to-CB charge transfer, minimized recombination, and extended electron lifetimes. The alignment of energy levels further validated the efficient electron injection from Aux LUMO to the TiO_2_ CB, positioning these NCs as more effective sensitizers than traditional plasmonic structures.

Expanding on structural stabilization, Kawawaki et al. addressed the challenge of NC aggregation post-ligand removal by designing a Cr_2_O_3_-encapsulated system using Au_25_(PET,p-MBA)_18_ NCs supported on BaLa_4_Ti_4_O_15_ [93]. Through controlled calcination at ~300 °C, the protective thiolate ligands were partially desorbed, exposing active gold surfaces while retaining NC size and structure. Subsequently, UV-induced deposition of a thin Cr_2_O_3_ layer created a stabilizing shell around the AuNCs, preventing sintering and preserving catalytic performance. This system demonstrated efficient and stable hydrogen and oxygen evolution with a 2:1 H_2_:O_2_ stoichiometry, and retained its photocatalytic activity over extended irradiation cycles. The optimized interfacial engineering and ligand desorption mechanism were supported by DIP-MS, EXAFS, and TEM analyses, establishing a model for the transition from ligand-protected to heterogeneous active catalysts. While Kawawaki et al. [93] relied on an oxide semiconductor support, Huang et al. developed a distinct MOF-based system [94]. They proposed an elegant in situ auto-reduction method to generate ultrasmall Pt clusters (1–2 nm) within the pores of MIL-125-NH_2_ modified by formaldehyde. The amino groups were converted to –NH–CH_2_OH, which served as internal reductants to reduce Pt^2+^ precursors into metallic Pt^0^ without external agents. This confinement strategy ensured high Pt dispersion, minimized aggregation, and optimized the metal–support interface. The resulting Pt(1.5)/MIL-125-NH-CH_2_OH catalyst achieved a hydrogen evolution rate of 4496.4 µmol·g^−1^·h^−1^ under visible light, approximately 31 times higher than the bare MOF, confirming the synergistic role of Pt NCs in accelerating charge transfer and enhancing photocatalytic efficiency. Using an alternative strategy for single-atom catalysis, another study employed thermal decomposition of Pt_5_(GS)_10_ NCs on multi-armed CdS nanorods, forming atomically dispersed Pt atoms coordinated with surface sulfur atoms [95]. These Pt–S_4_ sites acted as electron sinks, rapidly accepting photogenerated electrons and promoting spatial charge separation. Ultrafast transient absorption spectroscopy and DFT calculations confirmed that the Pt–S_4_ sites exhibited optimal hydrogen adsorption energetics, accounting for the high catalytic activity. The system delivered an impressive H_2_ evolution rate of 13.0 mmol·g^−1^·h^−1^ with 25.08% quantum efficiency at 400 nm, setting a benchmark for NC-derived single-atom hydrogen evolution reaction catalysts.

On the covalent organic frameworks (COF) front, Li et al. designed a π-conjugated 2D COF (PY-DHBD-COF) with adjacent hydroxyl and imine-N coordination sites that selectively adsorbed PtCl_6_^2−^ ions for in situ photoreduction into ultrasmall Pt clusters [96]. This structural design enabled site-specific anchoring of Pt and promoted electron transfer from the COF to Pt domains upon light irradiation. At optimal loading (3 wt% Pt), the system achieved a remarkable hydrogen evolution reaction rate of 71,160 µmol·g^−1^·h^−1^ and demonstrated 8.4% apparent quantum yield at 420 nm, along with 60 h operational stability and uniform Pt dispersion. DFT simulations validated the strong Pt binding energy and electron transfer pathways. Lastly, Bootharaju et al. demonstrated the catalytic superiority of bimetallic core–shell clusters [Au_12_Ag_32_(SePh)_30_]^4−^ electrostatically anchored on oxygen-deficient TiO_2_ for hydrogen evolution [97]. Compared to homometallic [Ag_44_(SePh)_30_]^4−^, the Au-doped system exhibited improved HOMO alignment, more effective charge separation, and superior NIR absorption. These characteristics enabled a hydrogen evolution rate of 6810 µmol·g^−1^·h^−1^, six times higher than the Ag-only system. XPS and DFT analyses confirmed that the substitution of Au in the cluster core induced favorable band shifts, enhancing electron transfer from TiO_2_ to the clusters and improving hydrogen evolution reaction performance under simulated sunlight. The system showed high photostability, retaining 90% of its activity after 16 h and minimal degradation over four months.

Altogether, atomically precise metal NCs play a crucial role in boosting photocatalytic water splitting through tailored structural integration, charge transfer engineering, and catalytic site optimization. Whether functioning as photosensitizers, co-catalysts, or single-atom active centers, NCs offer unique opportunities for enhancing charge separation, improving redox kinetics, and expanding light absorption. Moving forward, further innovation is expected in hybrid systems that combine the quantum-defined properties of NCs with porous hosts such as MOFs or COFs, or with oxygen-deficient semiconductors for synergistic light harvesting and catalysis. Key challenges remain in scaling up synthetic methods for stable NC incorporation, suppressing metal leaching, and extending performance testing to real-world conditions beyond sacrificial agents. Nonetheless, the precise tunability and integration versatility of NCs continue to position them as key enablers in the design of efficient and durable solar-to-hydrogen conversion platforms.

#### 3.4.3. Other Reactions

##### Light-Driven Nitrogen Fixation

Photocatalytic nitrogen fixation aims to mimic the natural process, converting inert nitrogen gas from the air into usable ammonia (NH_3_) under mild conditions. Ammonia produced via this method is considered a clean fuel that could play a crucial role in a future carbon-neutral economy. Recent efforts to enable sustainable ammonia synthesis under ambient conditions have led to the development of metal NC-based systems capable of photocatalytic nitrogen fixation. Sun et al. pioneered the use of atomically precise Au_4_Ru_2_(PPh_3_)_2_(SC_2_H_4_Ph)_8_ NCs for the photocatalytic reduction in dinitrogen to ammonia under ambient conditions [98]. These clusters, deposited on oxygen-deficient TiO_2_ (TiO_2_-Ov), exhibited excitonic behavior and a well-defined hexahedral structure where Ru atoms served as the primary active sites for N_2_ activation, while the Au atoms contributed to charge delocalization. Upon visible light irradiation, charge carriers were generated both in the TiO_2_ support and within the Au–Ru cluster, resulting in electron transfer toward the Ru atoms and simultaneous water oxidation by TiO_2_ to supply protons. DFT and in situ DRIFT spectroscopy confirmed that NH_4_^+^ formed via a distal or enzymatic pathway, with the rate-limiting step being either NNH formation or N–N cleavage. This hybrid catalyst achieved an ammonia production rate of 44.5 µmol·g^−1^·h^−1^, outperforming both Au-only clusters and bare TiO_2_, and demonstrating a unique dual-site catalytic mechanism. This work represents one of the first examples of light-driven N_2_ fixation using non-plasmonic, excitonic metal clusters, offering atomic-level insight into heterojunction engineering for artificial nitrogen fixation.

##### Dye Degradation

**Figure 13:**
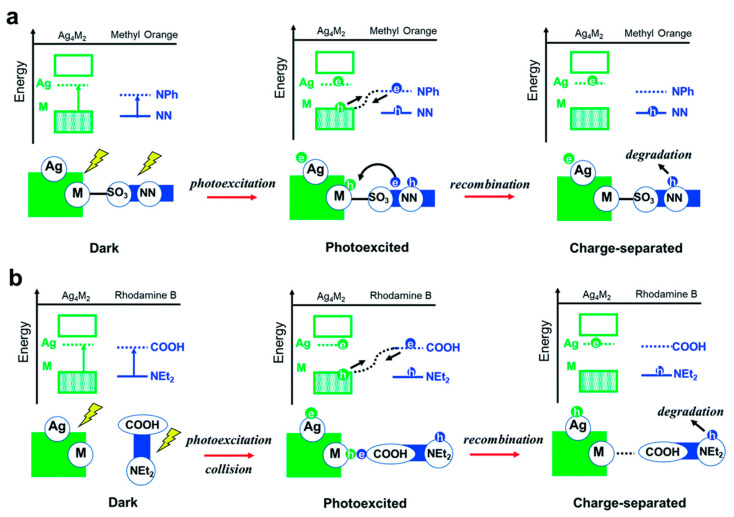
Schematic representation of the proposed photocatalytic degradation mechanisms of (**a**) methyl orange and (**b**) rhodamine B over Ag_4_M_2_(SPhMe_2_)_8_ NCs catalysts, where M = Ni, Pd, or Pt. Reprinted with permission from Ref. [99]. Copyright 2021 Royal Society of Chemistry.

The photocatalytic breakdown of organic pollutants remains a central environmental challenge, where NCs offer tunable reactivity and selectivity through metal-dopant engineering. Cheng et al. reported a series of Ag_4_M_2_(SPhMe_2_)_8_ NCs (M = Ni, Pd, Pt) supported on TiO_2_ for visible-light-driven photocatalytic degradation of methyl orange (MO) and rhodamine B (RhB) dyes [99]. The distorted hexahedral structure of these NCs exposed the dopant atoms, facilitating dye interaction and catalysis. Mechanistically, MO degradation followed a complexation-induced charge recombination pathway, initiated by electron transfer from dye to catalyst. In contrast, RhB degradation occurred via a collision-induced recombination route, where charge separation occurred upon dye–catalyst collision (Figure 13).

The electronic structure of the clusters, particularly the energy alignment between dopant HOMOs and Ag-based LUMOs, dictated reaction efficiency. Ag_4_Pd_2_ exhibited the fastest MO degradation (18 min), while Ag_4_Ni_2_ led in RhB breakdown, demonstrating substrate-specific catalytic behavior. All systems showed superior performance over pristine TiO_2_ and maintained high structural stability during catalysis. This study established a direct link between dopant selection, charge transfer dynamics, and selective dye degradation, highlighting NCs as tunable platforms for photocatalytic wastewater treatment.

##### Selective H_2_O_2_ Generation

Beyond hydrogen, the photocatalytic generation of hydrogen peroxide has emerged as a valuable reaction for green oxidation chemistry and disinfection, where surface-modified NCs provide a promising route. Xue et al. developed a hybrid photocatalyst based on glutathione-capped Au NCs covalently grafted with a cobalt-porphyrin ligand (Co-TCPP), enabling efficient and selective production of hydrogen peroxide from molecular oxygen [100]. The Au-Co-TCPP system functioned through a synergistic mechanism: visible-light excitation of the AuNCs initiated charge separation, with photogenerated electrons transferring to the Co(II) centers to reduce O_2_ to H_2_O_2_ via a 2e^−^ pathway, while holes oxidized water to O_2_, sustaining the redox cycle. Spectroscopic analyses (ESR, XPS, PL quenching) confirmed efficient electron transfer and minimized recombination. The hybrid yielded 235.93 mM H_2_O_2_ in 60 min with a turnover frequency of 3.33 h^−1^, more than double that of unmodified AuNCs. The system also displayed enhanced stability against H_2_O_2_ decomposition, attributed to GSH passivation. This work is the first to report ligand-engineered AuNCs for photocatalytic H_2_O_2_ production, demonstrating the potential of multifunctional ligand frameworks in controlling light harvesting, charge flow, and catalytic selectivity in NC-based platforms.

The studies presented in the catalysis section highlight the ability of metallic NCs to function as atom-efficient catalysts, enabling selective transformations in energy conversion, environmental remediation, and synthetic chemistry. Their well-defined atomic structures, abundant low-coordination sites, and ligand-tunable reactivity set them apart from conventional nanoparticle systems. To consolidate these advances, Table 5 summarizes representative examples of NCs employed in catalytic applications.

Despite the significant advances achieved in recent years, several limitations continue to constrain the practical deployment of NC-based catalysts. Catalytic performance is highly sensitive to subtle variations in cluster composition, ligand environment, and support interactions, which complicates reproducibility and hampers direct comparison across studies. Under operating conditions, NCs may undergo structural evolution, including ligand desorption, oxidation state changes, leaching, or sintering, processes that obscure the identification of true active sites and weaken structure–activity correlations. Trade-offs between activity and selectivity remain prevalent, particularly in complex multi-electron transformations, while the stability of NCs under realistic reaction feeds, such as those containing moisture or mixed gas streams, has not yet been systematically established. Addressing these challenges will require the development of standardized synthesis and purification protocols, accompanied by rigorous durability and recyclability testing under application-relevant conditions. Equally important is the rational design of ligands and supports that stabilize the atomic structure of the active sites while maintaining catalytic accessibility. Finally, advancing mechanistic understanding through the integration of in situ and operando spectroscopies with theoretical modeling will be essential for controlling dynamic active-site evolution. In parallel, efforts to establish scalable and environmentally sustainable synthetic routes, together with the incorporation of NCs into photoelectrochemical and continuous-flow architectures, are expected to accelerate the translation of laboratory-scale activity and selectivity into robust, device-level performance.

From a commercialization standpoint, the most promising near-term opportunities for NC catalysts lie in niche applications where atomic efficiency and light-driven activity offer clear benefits, such as on-site hydrogen peroxide generation, selective CO_2_-to-CO upgrading, photochemical fine-synthesis, and wastewater remediation. To progress beyond laboratory studies, NCs must be transitioned into immobilized, reactor-compatible forms through integration with scalable supports or continuous-flow/photoelectrochemical devices. Commercial viability depends on proving long-term durability under realistic feeds, developing closed-loop regeneration to minimize leaching, and establishing scalable, low-waste syntheses with robust quality control. Pilot-scale demonstrations accompanied by techno-economic and life-cycle assessments will be essential to evaluate cost, carbon intensity, and recyclability. Meeting these requirements could transform NCs into reliable, reactor-grade catalysts, enabling phased adoption from modular on-site systems toward larger-scale industrial deployment. Expansion of the field could come from systematically applying NCs to catalytic reactions already highlighted in laboratory studies but not yet tested under realistic conditions. Another promising direction is the design of ligand shells that are stable under photocatalytic or electrocatalytic bias to prevent desorption while maintaining access to active sites. These targeted advances would directly bridge the current laboratory findings with practical catalytic environments.

## 4. Conclusions and Perspectives

Metal NCs represent a unique class of materials that combine atomic-level precision with tunable optical, electronic, and catalytic properties, positioning them as powerful tools across multiple application domains. Advances in synthesis have enabled the development of NCs with precise size control, tailored emission wavelengths, enhanced stability, and improved reproducibility, creating a robust foundation for their integration into real-world technologies. In fluorescence imaging, NCs have shown deep tissue penetration, long fluorescence lifetimes, and strong biocompatibility, while in sensing, their tailored surface chemistry and responsive luminescence have enabled sensitive detection of ions, small molecules, and pathogens. Theranostics has emerged as a particularly promising direction, where NCs serve as both diagnostic and therapeutic platforms, enabling fluorescence-guided photothermal therapy, photodynamic therapy, gene delivery, and anti-inflammatory treatment with high spatiotemporal precision and minimal off-target toxicity. Additionally, catalytic applications have further highlighted their atom-efficient behavior and selective reactivity, especially in CO_2_ reduction and water splitting.

Despite these advances, several limitations must still be addressed. Synthetic reproducibility remains a major challenge, as small variations in reaction conditions can yield heterogeneous materials with inconsistent performance. Stability under complex biological or catalytic environments is another critical issue, with aggregation, ligand desorption, or structural evolution often compromising functionality. For biomedical applications, long-term toxicity, immunogenicity, and clearance pathways are insufficiently understood and require systematic investigation. In catalysis, durability under realistic feeds and large-scale operating conditions remains unproven. Overcoming these challenges will require standardized, scalable synthesis and purification methods, rational ligand and support engineering to stabilize active sites, and expanded use of in situ and operando spectroscopies to link structure, dynamics, and function.

From a commercialization perspective, translation is progressing but remains at an early stage. NC-based sensors are closest to deployment in environmental and food safety monitoring, yet require systematic validation under regulatory frameworks such as IVD and CLIA. In biomedicine, imaging and theranostic applications show strong potential but demand extensive long-term toxicology and pharmacokinetic studies before clinical use. Catalytic applications may reach industry sooner in niches such as on-site H_2_O_2_ production, wastewater remediation, and selective CO_2_ upgrading, provided that scalable synthesis, durability under realistic feeds, and pilot-scale techno-economic validation are achieved.

Overall, metal NCs stand at the interface between fundamental science and technological translation. With continued progress in synthesis, mechanistic understanding, and validation under practical conditions, they hold strong promise as next-generation materials for healthcare, environmental monitoring, and sustainable energy.

## Figures and Tables

**Table 1:** Summary of metal NCs synthesized via emerging methodologies.

Sample	Capping Ligand	Synthesis Method	Time	λ_exc_/λ_em_(nm)	Ref.
His-AuNCs	histidine	Microwave assisted	30 min	380/471	[19]
His-AuNCs	histidine	Microwave assisted	30 min	400/475–520	[20]
GSH-AuNCs	glutathione	Microwave assisted	20 min	405/610–800	[21]
His-AgNCs	histidine	Microwave assisted	8 min	356/440	[22]
Pep-CuNCs	pepsin	Microwave assisted	36 min	349/409	[23]
Ag_25_	(1,5-bis-(diphenylphosphino)pentane)	Photochemical assisted	24 h	588/n.r.	[24]
Ag_12_Cu_7_	(4-t BuPhC≡C)_14_(Dpppe)_3_	Photochemical assisted	24 h	345/665	[25]
Ni_10_(4-MePhS)_20_ Ni_11_(PhS)_22_ Pd_9_(PhS)_18_ Pd_10_(PhS)_20_	phenyl disulfide radicals	Photochemical assisted	8 h	467/n.r.	[26]
Cu(acac)_2_	monoethanolamine	Photochemical assisted	180 min	390/490	[27]
NAC-CuNCs	N-acetyl-L-cysteine	Sonochemical assisted	15 min	340/630	[29]
Ni/Ti_3_C_2_T_x_	Ti_3_C_2_Tx MXene	Sonochemical assisted	3–7 h	n.r.	[30]
PPh_4_[Au_13_(TBBT)_4_ (Dppe)_4_]Br_2_	phosphine (Dppe) ligands	Catalytic assisted	10 h	600/n.r.	[31]

* n.r.—not reported.

**Table 2:** Summary of metal NCs employed for imaging applications.

Sample	Capping Ligand	λ_ex_/λ_em_ (nm)	QY (%)	Imaging Model	Ref.
FA-BSA-AuNCs	bovine serum albumin	530/670	6	targerted in vitro imaging of NIH:OVCAR-3 cells	[40]
MUC1-AuNCs	thiolated MUC1 aptamer	420/655	4.2	targerted in vitro imaging of 4T1 cells	[41]
LAuNC	modified lycosin-I peptide	347/782	9.1	targerted in vitro imaging of 4T1 cells	[42]
GS-AuNPs	gluthatione	350.730–800	-	ex vivo imaging of kidney, brain, and small intestine tissue	[43]
BSA-AuNCs	bovine serum albumin	810TPE/655	6	ex vivo imaging of tissue-mimicking phantom	[44]
BSA-AuNCs	bovine serum albumin	820TPE/670	-	ex vivo imaging of tissue-mimicking phantom	[45]
GSH-AuNCs	gluthatione	405/610–800	9.9	ex vivo imaging of tissue-mimicking phantom	[21]
CD-Au NCs	thiolated cyclodextrin	808/1050	0.11	in vivo imaging of BALB/c nude mice bearing MCF-7 breast tumors	[46]
AS1411-AuNPs	Phosphorothioate-modified AS1411 DNA aptamer	808–980/1030	-	in vivo imaging of BALB/c mice bearing 4T1 breast cancer xenografts	[47]
TPPTS-AuNPs	triphenylphosphine-3,3′,3″-trisulfonic acid	808/1026	-	in vivo imaging of BALB/c mice with early-stage kidney injury	[48]
Au_7_Cd_1_-MHA/MPA	6-mercaptohexanoic acid (MHA) and 3-mercaptopropionic acid (MPA)	808/1000–1050	-	in vivo imaging of C57BL/6 male mice’ vessels	[49]
Au_25_(SG)_18_	gluthatione	730–800/850–950 and 1150–1400	-	in vivo imaging of healthy mice and mice with radiation-induced intestinal injury	[50]

**Table 3:** Summary of metal NCs employed for theranostics applications.

Sample	λ_ex_/λ_em_ (nm)	Biological Model	Therapeutic Mechanism	Performance	Ref.
ICG4−GS-Au25	808/820	female BALB/c mice bearing subcutaneous TUBO murine breast tumors	photothermal therapy triggered by LASER	complete tumor ablation	[51]
Min 23@AuNCs	808/1050	BALB/c mice with subcutaneous 4T1 breast tumors	Photodynamic therapy triggered by smartphone LED	~90% tumor growth inhibition; effective with low-cost light activation	[52]
AuNC–PTEN	-	BALB/c nude mice with HepG2 tumor	targeted therapeutic gene delivery	Strong tumor targeting (peak after 6h); significant tumor growth inhibition;	[53]
Au44MBA26-P NCs	808/1080–1280	mice with cattle-derived type II collagen immunization-induced rheumatoid arthritis	Anti-inflammatory and immunomodulatory	superior rheumatoid arthritis outcomes compared to methotrexate and non-phosphorylated Au44	[54]
Ag@PEG2000-HA NCs	-/600–800	BALB/c mice with 4T1 tumors	reactive oxygen species-mediated mitochondrial apoptosis	Early tumor signal; robust tumor inhibition; survival extended to 47–73 days vs. 26–50 days (control)	[55]
Au–Gd NCs	808/ >1000	BALB/c mice with subcutaneous TUBO breast tumors	photothermal therapy triggered by LASER	3× longer survival compared to controls; significant tumor volume reduction	[56]
Au_n_NCs-DPA	-	- computational	Drug delivery feasibility	solvation lowers binding energies; predicted facile release from Au surfaces	[57]

**Table 4:** Summary of metal NCs employed for sensing applications.

Sample	Analyte	Detection Strategy	Linear Range	Limit of Detection	Real Samples Performance	Ref.
GSH-Au NCs	Cobalt ion (Co^2^⁺)	Fluorescence quenching	2.0–50.0 µM	0.124 µM	102.8–108.3%	[60]
BSA-CuNCs	Ferric ion (Fe^3^⁺)	Fluorescence quenching	0.2–2.4 µM	10 nM	93.8–104.0%	[61]
MMI-CuNCs	Silver ion (Ag^+^)	Fluorescence quenching	0.025–50 µM	6.7 nM	97.0–104.0	[62]
11-MUA-AuNCs	Cadmium ion (Cd^2+^); Zinc ion (Zn^2+^); Copper ion (Cu^2+^)	Fluorescence enhancement for Cd^2+^ and Zn^2+^; Fluorescence quenching for Cu^2+^	Cd^2+^: 0.01–2.5 µM Zn^2+^: 0.025–5.0 µM Cu^2+^: 0.05–10 µM	Cd^2+^: 0.012 µM Zn^2+^: 0.016 µM Cu^2+^: 0.026 µM	Cd^2+^: 87.74–100.24% Zn^2+^: 91.51–103.18% Cu^2+^: 98.71–101.16%	[63]
CASE-AuNCs	Copper ion (Cu^2+^) Mercury ion (Hg^2+^)	Fluorescence quenching	Cu^2+^: 0–7 µM Hg^2+^: 0–14 µM	Cu^2+^: 14.78 nM Hg^2+^: 35.21 nM	Cu^2+^: 96.4–99.4% Hg^2+^: 96.3–98.9%	[64]
BSA-AuNCs	Copper ion (Cu^2+^)	Fluorescence quenching	-	5 µM	-	[65]
His-AuNCs	Ferrous ion (Fe^2+^) Ferric ion (Fe^3+^)	Fluorescence quenching	9−97 µM	3.2 µM	102.0–105.4%	[19]
AuNCs	Carbendazim	Fluorescence resonance energy transfer turn-on	1−100 µM; 150−1000 µM	0.83 µM; 37.25 µM	92.0–97.3%	[66]
DNA-AgNCs/Cu^2+^	glyphosate	Fluorescence turn-on	15–100 µg/L	5 µg/L	80.0–115.8%	[67]
DNA-AuNC	DNA methyltransferase	Fluorescence turn-off	0.5–40 U mL^−1^	0.178 U mL^−1^	92.5–110.5%	[68]
Fe_3_O_4_NPs@SiO_2_@AuNCs	microRNA-21 and microRNA-141	21: Fluorescence quenching 141: Fluorescence enhancement	21: 0.1 pM–10 nM 141: 0.1 pM–1 nM	21: 0.02 pM 141: 0.017 pM	21: 98.9–103% 141: 93.5–99.2%	[69]
SAN-CuNCs	Ascorbic acid	Fluorescence turn-on	25–400 µM	6.9 µM	94.8–105.3%	[70]
PEI/DTH@NiNCs	glutathione	Fluorescence enhancement	0–250 µM	0.007 µM	95.2–104.5%	[71]
Cu NCs@PP	Congo red	change in fluorescence color	0.5–160 µM	0.085 µM	97.2–110.8%	[72]
GSH-AuNCs	Staphylococcus aureus and Escherichia coli biofilms	Fluorescence enhancement	2.6 × 10^5^–6.7 × 10^7^ CFU/mL	1.7 × 10^5^ CFU/mL	-	[73]
aptamers@papain@AuNCs	*Escherichia coli* O157:H7	Fluorescence enhancement	10^1^–10^6^ CFU/mL	Pure culture: 39 CFU/mL	high sensitivity in ultra-high temperature (UHT), pasteurized, and raw milk (LODs ~500 CFU/mL)	[74]
3WJ/DNA-Ag/PtNCs	Salmonella typhimurium	Solution color change	2.6 × 10^2^–2.6 × 10^6^ CFU/mL	2.6 × 10^2^ CFU/mL	96.5–107.7%	[75]
Met-AuNCs	human papillomavirus	Cas12a-based electrochemiluminescence	1 pM–10 nM	0.48 pM	95.4–101.3%	[76]
CuNCs	hepatitis B virus DNA	Fluorescence quenching	0.5–100 pM	0.54 pM	99.1–102.1%	[77]
ssDNA-AuNCs	trypsin	Fluorescence turn-off	5 ng/mL–60 ng/mL	1.5 ng/mL	98.7%–103.5%	[78]
DNF@AuNCs	Aflatoxin B1	Fluorescence quenching	0.01–200 ng/mL	7 pg/mL	95.3–108.6%	[79]

**Table 5:** Summary of metal NCs employed for catalysis applications.

Sample	Co-Catalyst	Catalytic Reaction	Resulting Product	Performance	Ref.
Ag_25_(SPhMe_2_)_18_ NCs	-	CO_2_ reduction	CH_4_	28.95 µmol h^−^^1^ mg^−^^1^ CH_4_; 100% selectivity; 5.19% after 10h illumination	[87]
AuNCs	MOF	CO_2_ reduction	CO Trace of CH_4_ and H_2_	57.6 mmol g^−1^ h^−1^ CO over 5 h; maintains > 90% activity after 3 catalytic cycles;	[88]
Au_25_ NCs	BiOBr nanosheets	CO_2_ reduction	CO	Superior to previous BiOBr-based catalysts: 43.57 µmol CO g^−^^1^ h^−^^1^ (2.7× higher than unmodified BiOBr)	[89]
Cu_6_–NH NCs	-	CO_2_ reduction	CO	148.8 µmol g^−^^1^ h^−^^1^ CO superior to non-protonated ligand (Cu_6_N)-25.8 µmol g^−^^1^ h^−^^1^ CO; 5-cycle reuse with no significant loss of activity	[90]
Cu NCs	Zr-MOFs	CO_2_ reduction	HCOOH and CO	Cu NCs@MOF-801: 94 µmol h^−^^1^ g^−^^1^ HCOOH (66% selectivity) and 32 µmol h^−^^1^ g^−^^1^ CO; Cu NCs@UiO-66-NH_2_:128 µmol h^−^^1^ g^−^^1^ HCOOH (86% selectivity)	[91]
Cu_6_-NH_2_ NCs	-	CO_2_ fixation	oxazolidinones	1.54 g product at 97% yield	[82]
Ag_44_(SR)_30_	TiO_2_ NPs	Water splitting	H_2_	7.4 mmol h^−^^1^ g^−^^1^ (10 times higher than pure TiO_2_ and 5× higher than TiO_2_/Ag NPs); maintained 83% activity after 5 cycles	[90]
Au_x_@GSH NCs	PDDA layer with a CdTe shell over CdS nanowires	Water splitting	H_2_	4.42 mmol g^−^^1^ h^−^^1^ (14 times higher than CdS alone); increasing activity over multiple cycles	[91]
Au_x_@GSH NCs	TiO_2_ nanotube	Water splitting	-	outperformed their plasmonic counterparts in terms of photocurrent generation, charge carrier density, and applied bias photon-to-current efficiency	[92]
Au_25_(PET,p-MBA)_18_	BaLa_4_Ti_4_O_15_ or Cr(OH)_3_/BaLa_4_Ti_4_O_15_ semiconductors	Water splitting	H_2_	highly active heterogeneous catalysts; long-term stability	[93]
PtNCs	MIL-125-NH-CH_2_OH	Water splitting	H_2_	4496.4 µmol·g^−^^1^·h^−^^1^ (31 times higher than MIL-125-NH_2_ alone)	[94]
Pt_5_(GS)_10_ NCs	CdS nanorods	Water splitting	H_2_	13.0 mmol g^−^^1^ h^−^^1^ (6 times higher than CdS nanorods); 25.08% efficiency;	[95]
Pt NCs	π-conjugated 2D covalent organic framework (PY-DHBD-COF)	Water splitting	H_2_	71,160 µmol·g^−^^1^·h^−^^1^; 8.4% efficiency; stable for 60 h	[96]
Au_12_Ag_32_(SePh)_30_	TiO_2_ support	Water splitting	H_2_	6810 µmol·g^−^^1^·h^−^^1^; 0.96% efficiency; ~90% after 16 h operation	[97]
Au_4_Ru_2_NCs	TiO_2_ nanocrystals	N_2_ fixation	NH_3_	44.5 µmol·g^−^^1^·h^−^^1^	[98]
Ag_4_M_2_(SPhMe_2_)_8_ NCs (M is Ni or Pd or Pt)	TiO_2_ support	Methyl orange (MO) and Rhodamine B degradation (RhB)	-	Ag_4_Pd_2_/TiO_2_ Complete degradation of MO in 18 minutes; Ag_4_Ni_2_/TiO_2_: Fastest degradation of RhB	[99]
Au-Co-TCPP	-	O_2_ reduction	H_2_O_2_	235.93 mM in 60 min (2 times higher than bare AuNCs	[100]
